# Quantifying the Toll of Disuse: A Meta-Analysis of Skeletal Muscle Mass and Strength Loss Following Upper Limb Immobilization

**DOI:** 10.3390/jcm14248884

**Published:** 2025-12-16

**Authors:** Iván Cuyul-Vásquez, Felipe Ponce-Fuentes, Joaquín Salazar-Méndez, Alexis Sepúlveda-Lara, Luis Suso-Martí, Gabriel Nasri Marzuca-Nassr, Enrique Lluch, Joaquín Calatayud

**Affiliations:** 1 Departamento de Procesos Terapéuticos, Facultad de Ciencias de Salud, Universidad Católica de Temuco, Temuco 4813302, Chile; icuyul@uct.cl; 2 Faculty of Physiotherapy, University of Valencia, 46010 Valencia, Spain; 3 Escuela de Kinesiología, Facultad Medicina y Ciencias de la Salud, Universidad Mayor, Temuco 4810344, Chile; 4 Escuela de Ciencias del Deporte y Actividad Física, Facultad de Salud, Universidad Santo Tomás, Talca 3460000, Chile; 5 Doctorado en Ciencias mención Biología Celular y Molecular Aplicada, Universidad de La Frontera, Temuco 4811230, Chile; 6 Exercise Intervention for Health Research Group (EXINH-RG), Department of Physiotherapy, University of Valencia, 46010 Valencia, Spain; 7 Departamento de Ciencias de la Rehabilitación, Facultad de Medicina, Universidad de La Frontera, Temuco 4811230, Chile; 8 Physiotherapy in Motion Multispeciality Research Group (PTinMOTION), Department of Physiotherapy, University of Valencia, 46010 Valencia, Spain; 9 Pain in Motion Research Group (PAIN), Department of Physiotherapy, Human Physiology and Anatomy (KIMA), Faculty of Physical Education & Physiotherapy, Vrije Universiteit Brussel, 1050 Brussel, Belgium

**Keywords:** immobilization, disuse atrophy, muscular atrophy, muscle strength, muscle weakness

## Abstract

Enhancing our understanding of the specific characteristics that disuse-induced models should possess, based on the immobilized joint, could significantly enhance the effectiveness of research in this field. **Objective:** Our objective was to quantify the decrease in skeletal muscle mass and strength in humans subjected to upper limb disuse-induced models. **Methods:** PubMed, Scopus, Web of Science, Embase, LILACS, SPORTDiscus, CINAHL, and Epistemonikos databases were searched from inception to November 2025. Randomized controlled trials, cross-over clinical trials, or quasi-experimental studies performed in healthy adults ≥18 years old, subjected to an induced-disuse model to investigate the effects on skeletal muscle mass or strength were included. **Results:** Forty-five studies were included. Significant differences in skeletal muscle mass, equivalent to a small effect size (SMD = −0.453; 95% CI = −0.698 to −0.208; *p* < 0.001) and a total loss of 3.44% (estimated average daily decline = 0.16%) were observed after 21 days of immobilization. Skeletal muscle mass loss was heterogeneous between the arm (5.03%), forearm (1.56%), and hand (4.67%) muscles. Significant differences in strength, equivalent to a large effect size (SMD = −1.36; 95% CI = −1.69 to −1.02; *p* < 0.001) and a total loss of 18.06% (estimated average daily decline = 1.55%), were observed after 18 days of immobilization. Strength decreases were heterogeneous between the arm (16.67%), forearm (21.42%), and hand (10.46%) muscles. **Conclusion:** Based on evidence of very low certainty, upper limb disuse-induced models appear to induce a nonlinear loss of skeletal muscle mass and a suggested substantially more severe and rapid loss of strength in healthy young adults, with effects varying heterogeneously across different muscle groups. Despite data limitations, these estimates provide a basis for designing experimental countermeasures, though caution is warranted due to the heterogeneity of the findings.

## 1. Introduction

Various conditions throughout the lifespan, including aging, hospitalizations, surgical procedures, and traumas, such as fractures or sprains can induce disuse and impair skeletal muscle mass and strength. The influence of skeletal muscle mass and strength on health is well-established. Both lower muscle strength and muscle mass have been associated with poorer performance in basic and instrumental activities of daily living [1] and mortality [2]. For instance, it has been observed that a handgrip strength between 26 and 50 kg is an indicator of a lower risk of mortality from all causes [3].

Disuse-induced models, such as bed rest, limb suspension/immobilization, reduced physical activity, and microgravity, have enabled researchers to understand the mechanisms underlying the loss of skeletal muscle mass and strength in both animals and humans [4,5]. Disuse-induced models are frequently used in the study of countermeasures to reduce skeletal muscle mass and strength [4,6]. However, a high heterogeneity has been observed in the protocols used in healthy humans, in terms of disuse-induced models’ duration, immobilization devices (e.g., casts, splints, slings, or bandages), daily immobilization time, number of immobilized joints, and activities allowed/prohibited during disuse [7]. Understanding which models elicit more pronounced muscle atrophy or strength loss within a shorter timeframe and with enhanced safety measures would facilitate informed decision-making, thereby enhancing the external validity of studies employing disuse-induced models.

A systematic review with meta-analysis showed that disuse-induced models, using casts or braces applied to the lower limbs, lasting 7–14 days, decreased quadriceps skeletal muscle mass and strength by 8% and 21% in healthy participants, respectively [8]. In addition, the same systematic review demonstrated no differences in skeletal muscle mass and strength loss when comparing cast or brace disuse-induced models [8]. However, the effects of disuse-induced models of the upper limbs have been rarely investigated in the literature, and there are no meta-analyses published to date [7]. Given the morphological and functional differences with the lower limb, knowing the behavior of the strength and mass of the upper limb during disuse may be relevant to maximize the effectiveness of rehabilitation programs. Enhancing our understanding of the specific characteristics that disuse-induced models should possess, based on the immobilized joint, could significantly enhance the effectiveness of research in this field. Therefore, the aim of this systematic review with meta-analysis was to quantify the decrease in skeletal muscle mass and strength in humans subjected to upper limb disuse-induced models.

## 2. Methods

### 2.1. Protocol and Registration

The report of this research was carried out according to the Preferred Reporting Items for Systematic Reviews and Meta-analysis (PRISMA) and the recommendations of the JBI Manual for Evidence Synthesis [9,10]. The PRISMA 2020 checklist is available in Table S1 of the Supplementary Material. The protocol for this research was published in the International Prospective Register of Systematic Review (PROSPERO), with the registration number: CRD42023430345. No significant deviations regarding the outcome selection or statistical analysis plans occurred.

### 2.2. Eligibility Criteria

Studies that met the following inclusion criteria were eligible: (1) population: healthy humans over 18 years old; (2) intervention: studies in which at least one group had been subjected to an upper limb disuse-induced model (cast, sling, splint, rest bed, or other immobilization device) without any intervention to prevent skeletal muscle atrophy or muscle weakness during the immobilization period; (3) primary outcomes: skeletal muscle mass determined by the following measurements: Magnetic Resonance Imaging, Computed Tomography, Dual Energy X-ray Absorptiometry (DEXA), bioimpedance, ultrasonography, or anthropometry; upper limb muscle strength measured by dynamometry, force transducer, strain gauge, one-repetition maximum (1-RM), or a load cell; and (4) type of studies: randomized controlled trial (RCT), cross-over clinical trials, or quasi-experimental studies, published in English, Spanish, or Portuguese.

Studies were excluded if (1) they were conducted on animals; (2) they were conducted in patients subjected to immobilization due to disease, trauma, or surgery; and (3) they were published only in conference proceedings.

### 2.3. Information Sources

Two independent reviewers (AS-L and IC-V) searched PubMed, Scopus, Web of Science, Embase, LILACS, SPORTDiscus, CINAHL, and Epistemonikos from inception to 20 November 2025. Additionally, manual searches were performed on the references of the articles included in the database searches.

### 2.4. Search Strategies

Sensitive search strategies were performed. The search strategies for each database can be found in Table S2 (Supplementary Materials).

### 2.5. Study Selection Process

Two independent reviewers (AS-L and IC-V) performed the selection of the studies by using the Rayyan web application [11]. Search results in each database were stored in Research Information Systems (RIS) files that were then uploaded to Rayyan. After eliminating duplicates, the reviewers screened the articles by title and abstract. Studies that did not meet the eligibility criteria were discarded. Studies that were potentially eligible were retrieved for full-text review. Details of the studies discarded during the full-text review can be seen in Table S3 (Supplementary Materials). Discrepancies in the selection of articles were resolved by a third reviewer (GNM-N). The agreement rate between the reviewers was calculated using the Kappa statistic.

### 2.6. Data Collection Process and Data Items

Data extraction was performed independently by two reviewers (AS-L and I-CV). The following information of each study was extracted: characteristics of participants (sample size, age, level of physical activity, weight, height, and body mass index); disuse-induced model (type, time, protocol) and results pre- and post-experiments (skeletal muscle mass and muscle strength). Data were extracted exclusively from groups subjected to the disuse model alone, without concurrent countermeasures intended to mitigate the loss of skeletal muscle mass or strength (e.g., contralateral training, nutritional supplements, motor imagery).

### 2.7. Risk of Bias Assessment

Two independent reviewers (FP-F and JS-M) assessed the risk of bias by using the JBI Critical Appraisal Checklist for Quasi-Experimental Studies and Randomized Controlled Trials [12]. Discrepancies in the assessment of risk of bias were resolved by a third reviewer (IC-V).

### 2.8. Grading of Recommendation, Assessment, Development, and Evaluation

The certainty of evidence for skeletal muscle mass and muscle strength was assessed using the Grading of Recommendations Assessment, Development, and Evaluation (GRADE) approach [13]. The GRADE framework classifies evidence quality into four levels: high, moderate, low, or very low [13]. The results of the GRADE analysis are presented in Supplementary Table S4.

### 2.9. Effect Measures and Synthesis Methods

A quantitative synthesis was performed with Jamovi v.2.3 software [14]. Graphpad Prism v.8.0.2 was used to synthesize the results into graphs. In the case of missing data, the reviewers contacted the authors by email. ImageJ was used to estimate means and standard deviations when data were reported only in graphs [15]. A fixed or random effects model was used depending on the heterogeneity. Heterogeneity was assessed using I2 statistics. If the method for evaluating the results was homogeneous, the mean difference (MD) with a confidence interval of 95% was used. Otherwise, the standardized mean difference (SMD) was used to calculate the effect sizes. To calculate the SMD, the Sidik–Jonkman estimator with the Knapp and Hartung adjustment was used [16]. Effect sizes were considered trivial (SMD <0.2), small (SMD 0.2–0.5), medium (SMD 0.6–0.8), or large (SMD >0.8) [17]. Statistical significance was considered with a *p*-value < 0.05. To evaluate publication bias, the Egger regression asymmetry test was used, and a value of *p* < 0.10 was considered significant [18]. Subsequently, an analysis of moderating variables was carried out, based on the total time of the disuse-induced model (days) and the time of daily use of the immobilization device (hours). Studies conducted with a disuse-induced model using bed rest were excluded from the meta-analyses because there was no upper limb immobilization [19,20]. Furthermore, studies in which induced muscle damage was performed prior to disuse were excluded from the meta-analyses, because exercise prior to immobilization influences skeletal muscle mass and strength [21,22]. A subgroup analysis was performed based on the study design (RCT or quasi-experimental), device (cast or removable), body part (arm, forearm, and hand), muscle, and outcome measure. Subgroup analyses and meta-regressions were conducted for exploratory purposes; consequently, no adjustment for multiple comparisons was applied. The percentages of change in the pre-post-disuse-induced model (Δ_total_) and estimated average daily decline (↓_daily_) were calculated for the skeletal muscle mass and strength for the arm, forearm, and hand and for each of the outcome measures reported by the studies.

## 3. Results

### 3.1. Study Selection

**Figure 1:**
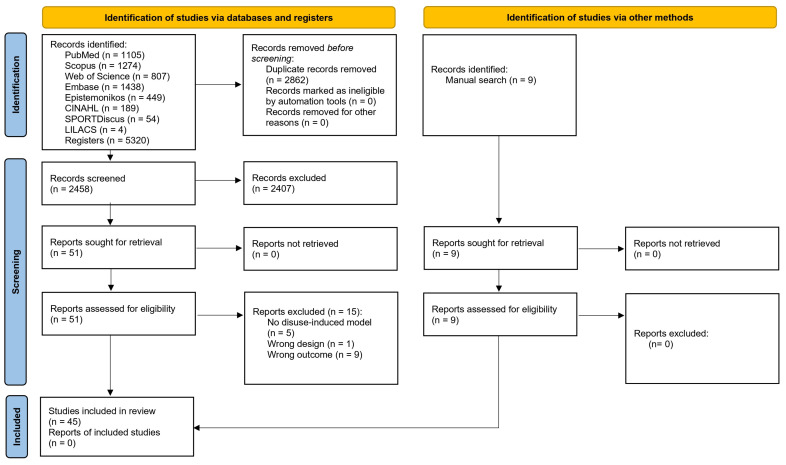
Flow diagram including searches of databases, registers, and other sources.

Electronic searches yielded a total of 5320 articles. In total, 2862 duplicates were detected and removed. Next, 2458 articles were reviewed using the title and abstract. Subsequently, a total of 48 articles were reviewed using the full text, of which 15 studies were excluded (see Table S3, Supplementary Materials). In addition, 9 potentially eligible studies were found through manual searches. Finally, 45 studies met the eligibility criteria and were included in this systematic review [19,20,21,22,23,24,25,26,27,28,29,30,31,32,33,34,35,36,37,38,39,40,41,42,43,44,45,46,47,48,49,50,51,52,53,54,55,56,57,58,59,60,61,62,63]. The agreement between reviewers in the study selection process was almost perfect (*k* = 0.97). Details of the study selection process are presented in Figure 1.

### 3.2. Study Characteristics

The characteristics of the included studies are summarized in Table 1. Regarding the study design, 42.2% (n = 19) were RCTs, 53.3% (n = 24) were quasi-experimental studies, and 4.7% (n = 2) were cross-over studies in clinical trials. In addition, 42.2% (n = 19), 42.2% (n = 19), and 14.0% (n = 6) of the articles studied changes in the strength or skeletal muscle mass in the arm, forearm, and hand, respectively. Only one study considered the arm and forearm to investigate the effects of disuse-induced models (2.2%) [35].

The sample of participants varied from 4 to 36 per study. A total of 449 participants, 267 men and 121 women, were subjected to a disuse-induced model. Four studies did not specify the number of men and women included in the immobilization groups [43,49,57,61]. The average age of the participants was 24.95 ± 8.62 years (20.3 to 66.6). Five studies included recreationally or physically active people [21,24,25,35,41,62], three studies included sedentary people [27,43,64], one study included normo-active people [51], and 33 studies did not report the physical activity level of the participants. One study reported the quantitative results of the Freiburg physical activity questionnaire (19.3 ± 10.3) but did not classify the physical activity of the participants [20]. Five studies reported the training experience of the participants with a mean of 2.83 months (2.0 to 3.9) [21,23,32,33,44].

Disuse-induced models used one (n = 27; 60.0%) or two devices to immobilize the upper limb (n = 16; 35.5%). Two studies (4.7%) did not use an immobilizing device, as the authors used a rest bed-based disuse-induced model [19,20]. Sling (n = 7; 16.3%), cast (n = 12; 27.9%), splint (n = 7; 15.5%), and brace (n = 1; 2.3%) were used in isolation whereas cast plus sling (n = 11; 25.6%), sling plus swathe (n = 4; 8.9%) or elastic bandage plus sling (n = 1; 2.3%) were used in combination. The shoulder, elbow, wrist, fingers, or thumb were the joints immobilized in the included studies. Immobilization was performed on the non-dominant (n = 40; 88.8%) or dominant (n = 1; 2.3%) upper limb or on one upper limb selected by randomization (n = 2; 4.7%). In two studies, no upper limb was immobilized [19,20]. On average, the studies reported using the disuse-induced models for 19.90 ± 14.69 days (0.5 to 90 days) with a daily time use of 21.24 ± 5.14 h (8 to 24). Thirty-four studies used the immobilizing device for 24 h, whereas in eleven studies the immobilizing device could be removed at some point. Three studies did not report whether the device could ever be removed [30,35,58]. Seventy-two percent of the studies included reported the specific immobilization angle. Studies targeting the elbow standardized the position at 90° of flexion. For studies targeting the wrist, the neutral position was the most frequent. Only one study utilized a position of slight wrist extension (15–30°) [48]. Studies involving the fingers employed very specific configurations depending on the target muscle, such as index finger flexion at 30–40° [54,55] or finger extension with thumb adduction [38].

### 3.3. Risk of Bias

**Figure 2:**
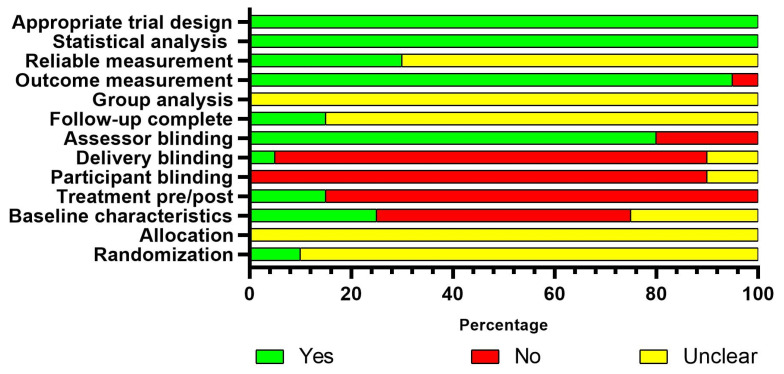
Assessment of risk of bias with the JBI critical appraisal checklist for randomized clinical trials.

**Figure 3:**
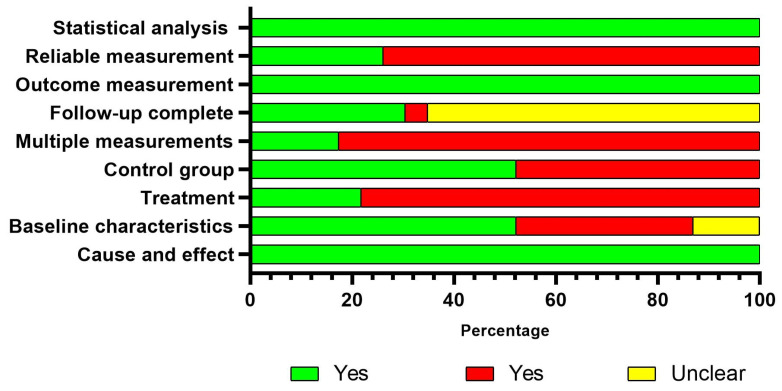
Assessment of risk of bias with the JBI critical appraisal checklist for quasi-experimental studies.

The mean critical appraisal score of the RCT was 4.90 ± 1.26. Three and eighteen RCT studies were considered at moderate and high risk of bias, respectively (Figure 2). The mean critical appraisal score of the quasi-experimental studies was 5.17 ± 1.43 points. Three, eleven, and ten quasi-experimental studies were considered at low, moderate, and high risk of bias, respectively (Figure 3). An individual evaluation of each study is available in the Tables S5 and S6 (Supplementary Materials).

### 3.4. Synthesis of Results

#### 3.4.1. Skeletal Muscle Mass

Nineteen studies provided a total of 29 datasets on skeletal muscle mass for meta-analysis. Based on the very low-certainty evidence, the overall pooled SMD estimate showed a small effect size, with statistically significant differences after the application of an upper limb disuse-induced model of 20.97 ± 7.03 days of immobilization (SMD = −0.453; *p* < 0.001; Δ_total_ = −3.44%; ↓_daily_ = −0.16%). Moderate statistical heterogeneity was observed (*I*^2^ = 49.60%, *p* = 0.011). Neither the rank correlation nor the regression test indicated any funnel plot asymmetry (*p* = 0.809 and *p* = 0.481, respectively). See the forest and funnel plot in Figures S1 and S2 (Supplementary Materials). According to the Cook’s distances, one study could be overly influential [58]. A sensitivity analysis was performed, and the study by Urso et al. (2006) [58], was consequently excluded. The sensitivity analysis reduced the statistical heterogeneity to “not important” (I^2^ = 16.30%, *p* = 0.970). However, the results continued to show a small effect size with statistically significant differences (SMD = −0.353; *p* < 0.001; Δ_total_ = −3.22%; ↓_daily_ = −0.14%). Inspection of the percentage changes suggested that anthropometric assessment (−1.13%) may be less sensitive than imaging methods (−4.10% to −4.64%). Consequently, a sensitivity analysis was conducted, excluding studies that estimated muscle mass using limb circumference. This sensitivity analysis revealed an increase in the magnitude of effect sizes and statistical heterogeneity (SMD = −0.56; *p* = 0.003; I^2^ = 58.34%; *p* = 0.001). See the forest and funnel plot in Figures S3–S6 (Supplementary Materials). The analysis of moderation showed that the number of days in the disuse-induced model (Estimate = −0.006; *p* = 0.837) and the time of daily use of the immobilizing device were not significant moderators of skeletal muscle mass loss (Estimate = 0.012; *p* = 0.458).

The subgroup analysis by study design demonstrated statistically significant differences in skeletal muscle mass with small effect sizes for the RCT (SMD = −0.25) and quasi-experimental studies (SMD = −0.59). Statistically significant differences were observed for the use of cast (SMD = −0.35) but not for the use of removable devices such as slings, brace, or splint (*p* = 0.067). With a small effect size (SMD = −0.43), the 21-day disuse-induced models were the most efficient at inducing skeletal muscle mass loss. The subgroup analysis by outcome measure demonstrated statistically significant differences in cross-sectional area (MD = −0.89 cm^2^), muscle thickness (MD = −0.13 cm), circumference (MD = −0.15 cm), and volume (MD = −0.96 cm^3^). Subgroup analysis by body part demonstrated statistically significant differences with small effect sizes in the skeletal muscle mass of the arm (SMD = −0.37) and forearm (SMD = −0.28). It was not possible to perform a subgroup analysis of the studies that evaluated skeletal muscle mass in the hand (N = 2). Small effect sizes were observed in the skeletal muscle mass of the biceps brachii plus brachialis (SMD = −0.36) and in the wrist flexor muscles (SMD = −0.59). Details of the subgroup analysis can be found in Table 2.

#### 3.4.2. Skeletal Muscle Strength

Thirty-three studies provided a total of 55 datasets on skeletal muscle strength for meta-analysis. Based on the very low-certainty evidence, the overall pooled SMD estimate showed a large effect size, with statistically significant differences after the application of upper limb disuse-induced models with 18.02 ± 9.50 days of immobilization (SMD = −1.36; *p* < 0.001; Δ_total_ = −18.06%; ↓_daily_ = −1.55%). Considerable statistical heterogeneity was observed (I^2^ = 82.76%, *p* < 0.001). See the forest and funnel plot in Figures S7 and S8 (Supplementary Materials). Both the rank correlation and the regression test indicated potential funnel plot asymmetry (*p* < 0.001 and *p* < 0.001, respectively). According to Cook’s distances, three studies could be overly influential. A sensitivity analysis was performed, and the studies by Clark et al. (2010), Clark et al. (2014), and Sayers et al. (2000) [28,31,53] were consequently excluded. Sensitivity analysis reduced the statistical heterogeneity, (I^2^ = 63.75%, *p* < 0.001). However, the results continued to show a large effect size with statistically significant differences (SMD = −1.14; *p* < 0.001; Δ_total_ = −16.60%; ↓_daily_ = −1.40%). See the forest and funnel plot in Figures S9 and S10 (Supplementary Materials). Meta-regression showed that the number of days in the disuse-induced model was not a significant moderator of the muscle strength loss (Estimate = 0.006; *p* = 0.714). However, the time of daily use of the immobilizing device was a significant moderator (Estimate = −0.07; *p* = 0.026).

The subgroup analysis by study design demonstrated statistically significant differences in skeletal muscle strength with large effect sizes for the RCT (SMD = −1.23) and quasi-experimental studies (SMD = −1.38). With a large effect size, the <10 days (SMD = −1.75) and 21 days (SMD = −1.54) were the most efficient at inducing skeletal muscle strength loss, respectively. Statistically significant differences were observed for the use of a cast (SMD = −1.40) and removable devices such as slings, brace, or splint (SMD = −1.30). Subgroup analysis by body part demonstrated statistically significant differences with medium-to-large effect sizes in the skeletal muscle strength of the arm (SMD = −0.79) and forearm (SMD = −1.82). No differences were observed in the hand muscles (*p* = 0.061). The subgroup analysis by outcome measure demonstrated statistically significant differences with medium-to-large effect sizes in isometric (SMD = −1.52) and concentric muscle strength (SMD = −0.77). It was not possible to perform a subgroup analysis of the studies that evaluated eccentric muscle strength (N = 2). Subgroup analysis by muscle demonstrated decreased strength with moderate-to-large effect sizes in the elbow extensors (SMD = −0.66), elbow flexors (SMD = −1.11), wrist extensors (SMD = −1.11), and wrist flexors (SMD = −2.21). No differences were observed in the first dorsal interosseus (*p* = 0.121). The grip strength decreased with a large effect size (SMD = −1.65). Details of the subgroup analysis can be found in Table 3.

## 4. Discussion

**Figure 4:**
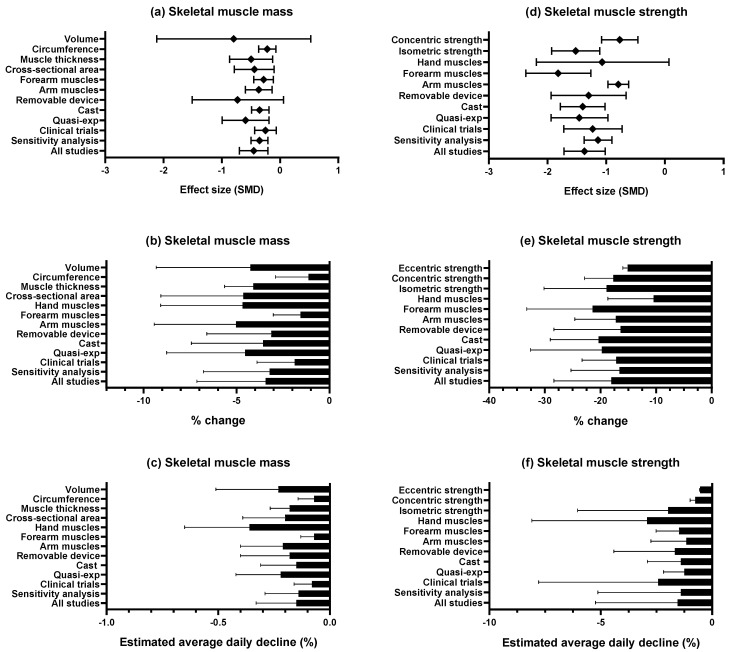
Effect sizes and percentage change in skeletal muscle mass and strength post upper limb disuse-induced model in healthy individuals.

Overall, our meta-analysis, based on very low-certainty evidence, indicates that upper limb disuse-induced models are effective in inducing decreases in skeletal muscle mass and strength in healthy young adults. However, the magnitude of these reductions varied heterogeneously across studies and subgroup analyses. Regarding skeletal muscle mass, we observed a small effect size corresponding to a total loss of 3.44% after 20.97 ± 7.03 days of disuse. Notably, regional differences were observed in skeletal muscle mass loss between the arm (−5.03%), forearm (−1.56%), and hand (−4.67%) (Figure 4). Conversely, a large effect size, equivalent to −18.06%, was observed for muscle strength loss after 18.02 ± 9.50 days of implementing an upper limb disuse-induced model. Similar to skeletal muscle mass, differences were observed between the muscle strength loss from the arm (−17.25%), forearm (−21.42%), and hand (−10.46%) (Figure 4). Generally, the rate of loss appeared to follow a nonlinear pattern; the estimated average daily decline was approximately 0.16% for skeletal muscle mass and 1.55% for strength. Importantly, these results may be underestimating clinical atrophy, as disuse-induced models do not account for the accelerated wasting induced by inflammation and metabolic stress in patients.

Our results are in line with those reported in a previous systematic review [7]. Campbell et al. (2019) estimated that the loss per day of skeletal muscle mass and strength in the upper limb during immobilization was 0.2% and 1.2–1.8%, respectively [7]. We were able to estimate the average daily decline and effect sizes with greater robustness and specificity due to the inclusion of a larger number of studies than Campbell et al. [7] (18 vs. 43 studies). In relation to lower limb disuse-induced models, a meta-analysis showed a decline from the baseline in lower limb skeletal muscle size and strength in healthy adults following single-leg disuse [8]. Preobrazenski et al. [8] reported a small effect size for quadriceps size loss with disuse-induced models (SMD = 0.41; n = 20 studies), similar to our findings in the upper limb. According to subgroup analyses by time, Preobrazenski et al. [8] showed that the effect sizes increased when longer duration disuse-induced models were used. The authors even observed significant differences with small effect sizes for disuse-induced models of fewer than 7 days [8]. Although we found small effect sizes with disuse models between 21 and 28 days, we did not find statistically significant differences for models with a duration of fewer than 10 days. In this sense, the percentages of change reflect these differences. For example, we found a 0.44% of skeletal muscle mass loss with a disuse-induced model in the upper limb while, in the lower limb, losses of 3 ± 1% have been observed with fewer than 7 days of immobilization [8].

Preobrazenski et al. [8] reported a moderate effect size for quadriceps strength loss with disuse-induced models. However, we found a large effect size for upper limb muscle strength loss. Factors such as a larger shortening position during the disuse-induced model [65] and differences in the cortical representation of the muscles between the upper and lower limb [66,67] could explain these differences. This could have important clinical implications, as the proportionally larger loss of muscle strength in the upper limb with disuse may affect the basic and instrumental activities of daily living. The upper limb disuse-induced models of fewer than 10 days showed a greater percentage decrease than those observed in the quadriceps muscle with models of ≤7 days duration (19.51% vs. 10.0%) [8]. This reinforces the need to develop countermeasures to the loss of muscle strength in the upper limb. Furthermore, our subgroup analysis showed that the muscles most affected by disuse-induced were the wrist flexors, with an estimated loss of 28.73%, equivalent to a large effect size. However, the total loss of strength with the induced disuse models in the elbow flexor/extensor muscles, wrist extensors, and handgrip strength varied between −14.25% and −19.96%.

Limb immobilization implies a drastic decrease in the mechanical stimuli that the skeletal muscle receives. The decrease in skeletal muscle mass with the disuse-induced models is mainly explained by a decrease in the rate of muscle protein synthesis, rather than by the increase in muscle degradation rates [68,69,70]. In disuse-induced models, protein synthesis rate reductions of 31 ± 12% have been observed in the lower limb [71]. This is mainly due to a decrease in the activation of the IGF-1–Akt-mTOR [72] and FAK-Akt-mTOR pathways [70]. However, muscle degradation pathways could be elevated in situations of reduced physical activity or hypercortisolemia [73,74]. Increased degradation pathways are characteristic of atrophy, due to disease, rather than disuse [4,68]. The decrease in skeletal muscle strength with the use of induced disuse models can be explained by several factors. It has been described that ∼79% of muscle strength loss could be explained by the decrease in skeletal muscle mass in the lower limb [75]. For example, from a structural point of view, longitudinal atrophy of the fascicles and muscle fibers [76], fatty infiltration [77,78], change in myosin content [79], decrease in muscle stiffness [69], and tendon stiffness could explain a lower strength [69]. Regarding neural adaptations, although a systematic quantitative analysis of neurophysiological variables was outside the scope of this review, the current evidence suggests that increased H-reflex amplitudes [42], decreased corticomuscular coherence [42], central muscular activation [29], decreased mean muscle discharge rate [55], prolongation of the corticospinal silent period [29], and neuromuscular junction instability [80] are neural factors that can also explain the decrease in strength following the disuse-induced models. Furthermore, disuse induces a decrease in glycogen reserves and insulin-stimulated leg glucose uptake, which could also explain the decrease in strength due to the alteration of energy metabolism [81].

Future research should strictly control confounding variables such as physical activity, training level, and diet, given their influence on muscle protein synthesis and anabolic resistance [82]. Ideally, skeletal muscle mass in a specific muscle should be assessed using gold-standard modalities such as magnetic resonance imaging (MRI) or computed tomography (CT) [83]. While ultrasound is an established reliable method for assessing skeletal muscle mass, it requires trained personnel, standardized muscle-specific protocols, and equipment with adequate resolution [84]. Conversely, techniques such as dual-energy X-ray absorptiometry (DXA), bioimpedance, and anthropometry are not recommended for studying skeletal muscle atrophy in disuse models due to their lower sensitivity in specific muscle segments. Regarding strength assessment, although most included studies used standardized dynamometry, immediate assessment following the removal of an immobilizing device may be confounded by joint stiffness, leading to underestimation. Therefore, it is advisable that future studies incorporate a recovery interval (e.g., 3–5 min) between device removal and assessment. During this period, an active or active-assisted mobilization protocol is recommended to reduce the stiffness. Furthermore, it is imperative that future studies explicitly report the immobilization angles (e.g., elbow at 90° or neutral wrist). The frequent absence of these data limits replicability, particularly given that the muscle length at which the limb is fixed directly modulates the structural adaptations to disuse. In addition, we observed a gap in the study of the effects of disuse in women and older people. We found preliminary evidence that women and older people have a different decrease in skeletal muscle mass and strength than men and young people during disuse-induced models [30,48,58]. This preliminary evidence is consistent with what was observed in the lower limb [85,86,87,88].

To ensure the validity of a disuse-induced model, future research should consider using a protocol that guarantees the decrease in skeletal muscle mass or strength. To this end, we propose a set of suggested parameters for experimental design based on our subgroup meta-analysis (Table 4). The recommendations are based on studies showing effect sizes ≥0.6 in skeletal muscle mass (N = 6) and ≥0.8 in muscle strength (N = 20) after the disuse-induced model. It is crucial to emphasize that these parameters are intended solely for designing experimental models in healthy volunteers and must not be interpreted as clinical guidelines for immobilization following fracture, surgery, or trauma. Researchers designing future upper limb disuse models might tailor the protocol to the specific muscle property and anatomical location. For instance, to induce changes in skeletal muscle mass, the combined use of a cast and sling appears effective, whereas removable devices or casts alone may be suitable for inducing experimental loss of muscle strength. Immobilization should target the joints spanning the muscle group of interest (e.g., the shoulder and elbow for arm muscles; the elbow, wrist, and fingers for forearm muscles). Durations of 21 days may be considered for detecting changes in skeletal muscle mass, while shorter periods (10–14 days) are sufficient for assessing strength. In addition, strict compliance with daily usage protocols is essential; for slings, a minimum daily wear time of 15–16 h may be considered, removing them only for sleeping or personal hygiene. Participants must be explicitly instructed to avoid any active use of the limb, such as lifting, pushing, or driving, during the study period.

### Limitations

The findings of these meta-analyses should be interpreted with caution for several reasons. Regarding the statistical validity, the large number of comparisons conducted in subgroup analyses increases the risk of Type I errors. Consequently, these findings should be considered as exploratory and hypothesis-generating, requiring confirmation in future studies. Furthermore, multiple effect sizes derived from single studies were treated as independent in the random-effects model, as multilevel analysis was not performed; this may have resulted in a slightly inflated precision and statistical significance of the pooled estimates. Sensitivity analyses were based on statistical influence, rather than methodological quality, due to the limited number of studies identified as low risk. Additionally, the reported effect size for strength loss should be interpreted carefully, as funnel plot asymmetry suggests potential publication bias and an overestimation of the true effect.

Methodological limitations regarding measurement and design must also be acknowledged. A relevant consideration is that 31% (n = 9) of the studies performed an indirect measurement of skeletal muscle mass using upper limb circumference or lean tissue mass. These methods possess lower validity and reliability, potentially leading to overestimation or underestimation of effect sizes [89,90]. Moreover, our results regarding the estimated average daily decline should be analyzed as descriptive averages rather than physiological kinetics, given that the skeletal muscle mass loss exhibited nonlinear behavior, and the model duration was not a significant moderator.

Limitations regarding the study population and external validity are critical. Due to the scarcity of data, it was not possible to perform a meta-analysis of skeletal muscle mass in hand muscles, eccentric muscle strength, or measurements in older adults; notably, the study by Urso et al. (2009) was excluded from sensitivity analyses [58]. Consequently, our results primarily reflect data derived from healthy young adults. Extrapolation to specific populations—such as geriatric patients, those with frailty, or clinical cases involving trauma and inflammation—is uncertain and likely underestimates the atrophy occurring in pathological states. Furthermore, insufficient data prevented the analysis of moderator variables regarding sex, diet, BMI, and physical activity level. Specifically, the impact of baseline fitness and diet remains unknown; considering that trained individuals and those with higher protein intake may exhibit different atrophy kinetics; this lack of stratification is a significant limitation.

Finally, this review focused on physiological surrogate outcomes. We did not synthesize data on functional disability, participation, or quality of life. Therefore, the findings of this systematic review should not be used in isolation to inform immobilization or rehabilitation protocols, particularly given that patient-reported outcomes and functional measures were not assessed. Clinical decision-making must be guided by individual patient characteristics, comorbidities, and functional goals.

## 5. Conclusions

While overall muscle mass shows an estimated decline of approximately 3.44% over nearly 21 days of disuse, our analysis suggests notable disparities emerge across specific segments of the limb in healthy young adults. For instance, the arm appears to register a higher loss (estimated at −5.03%) compared to the forearm (−1.56%) and hand (−4.67%). In contrast, the impact on muscle strength seems more pronounced, with a suggested decrease of approximately −18.06%, following roughly 18 days of disuse. Again, variations in strength loss are evident across different regions, with the forearm exhibiting the most substantial estimated decline (−21.42%), followed by the arm (−16.67%) and the hand (−10.46%). These results suggest a potential loss in skeletal muscle mass and strength in the hand, though these findings are based on limited data and warrant cautious interpretation. Given the very low certainty of evidence, high heterogeneity, and risk of bias in the included studies, these estimates should be interpreted with caution. Despite data limitations, these estimates provide a basis for designing experimental countermeasures.

## Figures and Tables

**Table 1:** Characteristics of studies.

Author and Year	Body Part	Design	Characteristics of the Participants						Characteristics of Disuse-Induced Models						
			n	IG n	Age (years)	M	F	PA or Training Experience	Device	Limb Immobilized	Time (days)	Daily Time Use (hours)	Remove Device	Joint Immobilized	Angle Joint Position
**Bostock et al., 2017a** [24]	Arm	RCT	24	8	26 ± 6.7	2	6	Recreationally active	Sling	Nondominant	14	9	Sling	Shoulder and elbow	90° elbow flexion
**Bostock et al., 2017b** [25]	Arm	RCT	24	8	26 ± 6.7	2	6	Recreationally active	Sling	Nondominant	14	9	Sling	Shoulder and elbow	90° elbow flexion
**Boer et al., 2008** [19]	Arm	CES	10	10	22 ± 2.2	10	0	NR	Bed rest	NA	35	24	NA	NA	NA
**Chen et al., 2005** [21]	Arm	RCT	33	11	21 ± 3.1	11	0	Physically active	Cast	Nondominant	4	24	NO	Shoulder and elbow	NR
**Chen et al., 2023** [26]	Arm	CES	24	12	23 ± 2.1	12	0	Sedentary	Cast + sling	Nondominant	21	24	Sling	Shoulder and elbow	90° elbow flexion
**Chen et al., 2023** [27]	Arm	CES	36	12	22.7 + 1.7	12	0	Sedentary	Cast + sling	Nondominant	14	24	Sling	Shoulder and elbow	90° elbow flexion
**Gaffney et al., 2021** [35]	Arm and forearm	RCT	24	12	21 ± 0.6	12	0	Recreationally active	Sling + swathe	Dominant	3	24	NO	Shoulder, elbow, and wrist	NR
**Johnston et al., 2009** [39]	Arm	Crossover	7	7	22	7	0	NR	Plaster Cast	Randomized	7	24	NO	Elbow	90° elbow flexion
**Karolczak et al., 2009** [40]	Arm	CES	18	7	30 ± 7.6	7	0	NR	Plaster Cast	Nondominant	14	24	NO	Elbow	90° elbow flexion
**Magnus et al., 2010** [44]	Arm	CES	25	8	20 ± 1.8	2	6	2.0 ± 3.9 months	Sling + swathe	Nondominant	28	13	Sling + swathe	Shoulder and elbow	90° elbow flexion
**Miles et al., 2005** [46]	Arm	CES	31	16	21 ± 3	6	10	NR	Sling + swathe	Nondominant	21	16	Sling + swathe	Shoulder and elbow	90° elbow flexion
**Parcell et al., 2000** [51]	Arm	CES	6	6	23 ± 1	6	0	Normo-active	Sling	Nondominant	28	16	Sling	Shoulder and elbow	90° elbow flexion
**Pearce et al., 2013** [52]	Arm	RCT	28	9	25 ± 8.7	4	5	NR	Sling	Nondominant	21	15	Sling	Shoulder and elbow	90° elbow flexion
**Sayers et al., 2000a** [53]	Arm	RCT	26	9	20 ± 0.4	9	0	NR	Cast + sling	Nondominant	4	24	Sling	Shoulder and elbow	90° elbow flexion
**Semmler et al., 1999** [56]	Arm	CES	16	12	18–45	6	6	NR	Cast + sling	Nondominant	28	24	Sling	Shoulder, elbow, and wrist	NR
**Valdes et al., 2021** [59]	Arm	RCT	30	10	23 ± 5.0	6	4	NR	Sling	Nondominant	28	8	Sling	Shoulder and elbow	90° elbow flexion
**Vaughan, 1989** [60]	Arm	CES	6	6	31.2	4	2	NR	Plaster Cast + sling	Nondominant	14	24	Sling	Shoulder, elbow, and wrist	90° elbow flexion
**Yue et al., 1997** [61]	Arm	CES	10	10	19–27	NR	NR	NR	Cast + sling	Nondominant	28	24	Sling	Shoulder and elbow	90° elbow flexion
**Zainuddin et al., 2005** [22]	Arm	CE	10	10	23 ± 4.2	5	5	NR	Splint + sling	Randomized	4	16	Sling	Shoulder and elbow	90° elbow flexion
**Andrushko et al., 2018** [23]	Forearm	RCT	16	8	23 ± 5	2	6	2.9 ± 4.3 months	Cast	Nondominant	28	24	NO	Wrist, thumb, and proximal interphalangeal joints	Wrist in neutral position
**Clark et al., 2014** [28]	Forearm	CES	29	15	21 ± 3.5	8	7	NR	Splint + sling	Nondominant	28	24	Sling	Wrist and fingers	Wrist in neutral position
**Clark et al., 2008** [29]	Forearm	CES	19	10	21 ± 0.5	5	5	NR	Splint	Nondominant	21	24	Sling	Wrist and fingers	Wrist in neutral position
**Clark et al., 2009** [30]	Forearm	CES	10	10	18–29	5	5	NR	Splint	Nondominant	21	24	NR	Wrist and hand	Wrist in neutral position
**Clark et al., 2010** [31]	Forearm	CES	20	11	20 ± 0.4	6	5	NR	Splint	Nondominant	21	24	Splint	Wrist and fingers	NR
**Farthing et al., 2009** [32]	Forearm	CES	30	10	22 ± 2.8	2	8	2.5 ± 3.9 months	Cast	Nondominant	21	24	NO	Wrist, thumb, and proximal interphalangeal joints	Wrist in neutral position
**Farthing et al., 2011** [33]	Forearm	CES	14	7	22 ± 4.4	1	6	3.9 ± 1.6 months	Cast	Nondominant	21	24	NO	Wrist, thumb, and proximal interphalangeal joints	Wrist in neutral position
**Homma et al., 2009** [36]	Forearm	RCT	15	7	20–29	7	0	NR	Cast + sling	Nondominant	21	24	Sling	Elbow and wrist	90° elbow flexion
**Homma et al., 2015** [37]	Forearm	RCT	34	7	22 ± 3.0	7	0	NR	Cast + sling	Nondominant	21	24	Sling	Elbow and wrist	90° elbow flexion
**Kitahara et al., 2003** [41]	Forearm	CES	6	6	21 ± 1.4	6	0	Physically active	Cast + sling	Nondominant	21	24	Sling	Elbow and wrist	NR
**Lundbye-Jensen-Nielsen et al., 2008** [42]	Forearm	CES	10	10	24 ± 6	6	4	NR	Cast	Nondominant	7	24	NO	Wrist and fingers	Wrist in neutral position
**MacIntyre et al., 2001** [43]	Forearm	RCT	9	9	24 ± 44	NR	NR	Sedentary	Plaster Cast	Nondominant	42	24	NO	Wrist	NR
**Matsumura et al., 2008** [45]	Forearm	RCT	10	5	23 ± 3.3	5	0	NR	Cast + sling	Nondominant	21	24	Sling	Elbow, wrist, and fingers	NR
**Motobe et al., 2004** [47]	Forearm	RCT	14	8	23 ± 2.6	8	0	NR	Cast	Nondominant	21	24	NO	Elbow, wrist, and fingers	NR
**Newsom et al., 2003** [48]	Forearm	RCT	18	8	13–30	0	8	NR	Cast	Nondominant	10	24	NO	Wrist	Wrist in 15–30° extension
**Ohmori et al., 2010** [50]	Forearm	RCT	21	7	22 ± 2.9	7	0	NR	Cast + sling	Nondominant	21	24	Sling	Elbow, wrist, and fingers	NR
**Ulloa-Escalante et al., 2022** [57]	Forearm	RCT	14	7	18 ± 0.9	NR	NR	NR	Sling	Nondominant	6	16	Sling	Shoulder and elbow	NR
**Rittweger et al., 2005** [20]	Forearm	RCT	25	9	32 ± 4.2	9	0	19.3 ± 10.3 Freiburg Questionnaire	Bed rest	NA	90	24	NA	NA	NA
**Fuglevand et al., 1995** [34]	Fingers	CES	11	11	24–45	8	3	NR	Splint	Nondominant	21	24	Splint	Thumb and index finger	Flexed position
**Inada et al., 2015** [38]	Fingers	RCT	30	10	29 ± 4.2	10	0	NR	Elastic bandage + sling	Nondominant	0.5	12	Sling	All fingers	Fingers extended; thumb adducted.
**Ngomo et al., 2012** [49]	Fingers	Crossover	11	11	26 ± 4.3	NR	NR	NR	Splint	Nondominant	4	24	NO	Wrist and fingers	NR
**Seki et al., 2001** [54]	Fingers	CES	7	7	21–22	7	0	NR	Cast	Nondominant	42	24	CAST	Middle and index finger and thumb	Index flexed 30–40°
**Seki et al., 2007** [55]	Fingers	CES	5	5	22–29	5	0	NR	Cast	Nondominant	7	24	NR	Middle and index finger and thumb	Index flexed 30–40°
**Urso et al., 2006** [58]	Fingers	CES	28	28	66 ± 1 20 ± 0.6	20	0	NR	Brace	Nondominant	14	24	NR	Thumb	NR
**Stock et al., 2025** [62]	Forearm	CES	30	20	22 ± 3.0	10	10	High-moderate activity	Splint	Non-dominant	7	24	NR	Wrist, thumb, and fingers	NR
**Carr et al., 2025** [63]	Arm	RCT	10	4	19 ± 0.5	0	4	NR	Sling + swathe	Non-dominant	28	10	Sling + swathe	Shoulder and elbow	90° elbow flexion

Abbreviations: NR: not reported; CES: quasi-experimental study; RCT: randomized controlled trial; IG: immobilization group; NA: not applicable. M: male; F: Female; PA: physical activity.

**Table 2:** Subgroup comparison of skeletal muscle mass during disuse-induced model.

Groups	Subgroup	% Change		Meta-Analysis						Heterogeneity	
		Per Model	Per Day	N Data	Effect Size Model	Intercept	SE	CI 95%	*p*	I^2^ (%)	*p*
**Design**	RCT	−1.87	−0.08	12	RE-SMD	−0.25	0.08	−0.44 to −0.07	0.013	7.78	0.975
	Quasi-exp.	−4.54	−0.22	17	RE-SMD	−0.59	0.19	−0.99 to −0.19	0.007	61.9	<0.001
**Device**	Cast	−3.57	−0.15	20	RE-SMD	−0.35	0.07	−0.49 to −0.19	<0.001	0	0.991
	Removable	−3.13	−0.18	9	RE-SMD	−0.73	0.34	−1.51 to 0.06	0.067	78.9	<0.001
**Body part**	Arm	−5.03	−0.21	13	RE-SMD	−0.37	0.11	−0.59 to −0.14	0.005	19.3	0.800
	Forearm	−1.56	−0.07	13	RE-SMD	−0.28	0.08	−0.45 to −0.11	0.003	5.6	0.991
	Hand	−4.67	−0.36	2 *	-	-	-	-	-	-	-
**Outcome**	CSA (cm^2^)	−4.64	−0.20	9	FE-MD	−0.89	0.25	−1.38 to −0.39	<0.001	0	0.931
	MT (cm)	−4.10	−0.18	5	FE-MD	−0.13	0.02	−0.19 to −0.06	0.005	0	0.924
	CIR (cm)	−1.13	−0.07	9	FE-MD	−0.15	0.05	−0.27 to −0.04	0.017	0	0.989
	VOL (cm^3^)	−4.26	−0.23	6	FE-MD	−0.96	0.09	−1.15 to −0.77	<0.001	62.7	0.020
**Muscle**	BB+B	−5.77	−0.23	6	RE-SMD	−0.36	0.16	−0.76 to 0.04	0.007	17.6	0.690
	WF	−3.62	−0.16	3	RE-SMD	−0.59	0.21	−1.51 to 0.34	0.112	12.3	0.580
**Disuse days**	≤10 days	−0.44	−0.09	3	RE-SMD	−0.07	0.05	−0.28 to 0.12	0.236	0.05	0.969
	21 days	−2.69	−0.13	15	RE-SMD	−0.43	0.10	−0.65 to −0.21	<0.001	17.9	0.852
	28 days	−5.08	−0.18	9	RE-SMD	−0.24	0.06	−0.37 to −0.10	0.003	1.3	0.999

Abbreviations: BB+B = biceps brachii plus brachialis; CI: confidence interval; CIR: circumference; CSA: cross-sectional area; FE: fixed effect; MD: mean difference; MT: muscle thickness; RCT: randomized clinical trial; RE: random effect; SE: standard error; SMD: standardized mean difference; VOL: volume; WF: wrist flexors. * Due to the limited number of studies, it was not possible to perform a meta-analysis of hand skeletal muscle mass and eccentric muscle strength.

**Table 3:** Subgroup comparison of skeletal muscle strength during disuse-induced model.

Groups	Subgroup	% Change		Meta-Analysis						Heterogeneity	
		Per Model	Per Day	N Data	Effect Size Model	Intercept	SE	CI 95%	*p*	I2 (%)	*p*
**Design**	RCT	−17.17	−2.42	24	RE-SMD	−1.23	0.24	−1.72 to −0.73	<0.001	81.2	<0.001
	Quasi-exp.	−19.76	−1.25	31	RE-SMD	−1.46	0.24	−1.94 to −0.97	<0.001	83.9	<0.001
**Device**	Cast	−20.35	−1.40	31	RE-SMD	−1.40	0.19	−1.78 to −1.02	<0.001	74.8	<0.001
	Removable	−16.41	−1.67	24	RE-SMD	−1.30	0.31	−1.94 to −0.66	<0.001	88.5	<0.001
**Body part**	Arm	−17.25	−1.16	20	RE-SMD	−0.79	0.08	−0.97 to −0.61	<0.001	16.7	0.941
	Forearm	−21.42	−1.48	27	RE-SMD	−1.82	0.19	−2.37 to −1.26	<0.001	83.76	<0.001
	Hand	−10.46	−2.92	6	RE-SMD	−1.07	0.45	−2.19 to 0.07	0.061	78.3	<0.001
**Outcome**	Iso	−18.9	−1.97	45	RE-SMD	−1.52	0.20	−1.93 to −1.12	<0.001	84.3	<0.001
	Con	−17.70	−0.76	8	RE-SMD	−0.77	0.13	−1.08 to −0.46	<0.001	15.9	0.773
	Ecc	−15.09	−0.54	2 *	-	-	-	-	-	-	-
**Muscle or action**	EE	−14.60	−0.76	6	RE-SMD	−0.66	0.19	−1.14 to −0.18	0.017	26.1	0.594
	EF	−18.31	−1.32	13	RE-SMD	−1.11	0.33	−1.81 to −0.39	0.005	82.9	0.010
	WE	−19.06	−1.55	5	RE-SMD	−1.11	0.32	−1.99 to −0.23	0.025	51.2	0.141
	WF	−28.73	−1.64	8	RE-SMD	−2.21	0.82	−4.15 to −0.27	0.031	93.7	<0.001
	FDI	−12.29	−4.14	4	RE-SMD	−0.42	0.20	−1.06 to 0.20	0.121	13.9	0.587
	HG	−15.69	−1.43	13	RE-SMD	−1.65	0.27	−2.23 to −1.06	<0.001	68.3	<0.001
**Disuse days**	≤10 days	−20.18	−5.18	9	RE-SMD	−1.75	0.52	−2.95 to −0.54	0.010	88.7	<0.001
	14 days	−11.93	−0.85	8	RE-SMD	−1.04	0.35	−1.87 to −0.21	0.021	71.01	<0.001
	21 days	−17.11	−0.81	20	RE-SMD	−1.54	0.25	−2.05 to −1.02	<0.001	77.13	<0.001
	28 days	−21.95	−0.78	12	RE-SMD	−1.08	0.47	−2.11 to −0.06	<0.040	90.06	<0.001
	>28 days	−23.00	−0.76	3	RE-SMD	−1.61	0.83	−5.19 to 1.96	0.192	82.9	<0.008

Abbreviations: CI: confidence interval; RCT: randomized clinical trial; RE: random effect; SE: standard error; SMD: standardized mean difference; EE: elbow extensors; EF: elbow flexors; WE: wrist extensors; WF: wrist flexors; FDI: first dorsal interosseus; HG: hand-grip; Iso: isometric contraction; Con: concentric contraction; Ecc: eccentric contraction. * Due to the limited number of studies, it was not possible to perform a meta-analysis of hand skeletal muscle mass and eccentric muscle strength.

**Table 4:** Suggested experimental parameters for upper-limb disuse-induced models in healthy individuals.

Characteristic	Disuse-Induced Model *	
	Arm Muscles	Forearm Muscles
**Immobilizer device**	For skeletal muscle mass only cast plus sling; for skeletal muscle strength removable device (sling) or cast.	For skeletal muscle mass only cast plus sling; for skeletal muscle strength removable device (splint, brace, bandage) or cast.
**Joint immobilized**	Shoulder and elbow.	Elbow and wrist; wrist and fingers.
**Joint position**	90° elbow flexion.	Wrist in neutral position.
**Time model**	For skeletal muscle mass: 21 days. For skeletal muscle strength: <10 to 14 days.	
**Daily time used**	Cast 24 h. Sling 16–15 h.	
**Device removal**	Sling removal for sleeping or bathing.	
**Instructions**	During the study, avoid using the upper limb to lift, push, pull, or hold objects, lifting weight, performing vigorous physical activity, or driving a car. It is optional to instruct the participant to consider the limb injured.	

* It was not possible to make recommendations on disuse-induced models of the hand due to the small number of published articles.

## Data Availability

Data are contained within the article.

## Supplementary Materials

The following supporting information can be downloaded at https://www.mdpi.com/article/10.3390/jcm14248884/s1, Table S1: PRISMA 2020 checklist. Table S2: Search strategies. Table S3: Studies excluded. Table S4: Grading of Recommendation, Assessment, Development and Evaluation (GRADE). Table S5: Risk of bias of clinical trials. Table S6: Risk of bias of quasi-experimental studies. Figure S1: Forest plot skeletal muscle mass. Figure S2: Funnel plot skeletal muscle mass. Figure S3: Sensitivity analysis. Forest plot skeletal muscle mass. Figure S4: Sensitivity analysis. Funnel plot skeletal muscle mass. Figure S5: Sensitivity analysis that includes only imaging studies. Forest plot skeletal muscle mass. Figure S6: Sensitivity analysis that includes only imaging studies. Funnel plot skeletal muscle mass. Figure S7: Forest plot skeletal muscle strength. Figure S8: Funnel plot skeletal muscle strength. Figure S9: Sensitivity analysis. Forest plot skeletal muscle strength. Figure S10: Sensitivity analysis. Funnel plot skeletal muscle strength.

## Abbreviations

The following abbreviations are used in this manuscript:

1-RM	One-repetition maximum
BMI	Body mass index
DEXA	Dual energy X-ray absorptiometry
MD	Mean difference
RCT	Randomized controlled trial
SMD	Standardized mean difference
Δ_total_	Percentages of change in the pre–post-disuse-induced model
↓_daily_	Estimated average daily decline
