# Supplementary material for: Quantifying the Toll of Disuse: A Meta-Analysis of Skeletal Muscle Mass and Strength Loss Following Upper Limb Immobilization
Source: J Clin Med. 2025 Dec 16;14(24):8884. doi: 10.3390/jcm14248884 (PMC12733654; doi:10.3390/jcm14248884)

Supplementary Materials

Table S1. PRISMA 2020 checklist

Section and Topic	Item #	Checklist item	Location where item is reported
**TITLE**			
Title	1	Identify the report as a systematic review.	Page 1
**ABSTRACT**			
Abstract	2	See the PRISMA 2020 for Abstracts checklist.	Page 1
**INTRODUCTION**			
Rationale	3	Describe the rationale for the review in the context of existing knowledge.	Page 2
Objectives	4	Provide an explicit statement of the objective(s) or question(s) the review addresses.	Page 2
**METHODS**			
Eligibility criteria	5	Specify the inclusion and exclusion criteria for the review and how studies were grouped for the syntheses.	Page 3
Information sources	6	Specify all databases, registers, websites, organisations, reference lists and other sources searched or consulted to identify studies. Specify the date when each source was last searched or consulted.	Page 3
Search strategy	7	Present the full search strategies for all databases, registers and websites, including any filters and limits used.	Page 3
Selection process	8	Specify the methods used to decide whether a study met the inclusion criteria of the review, including how many reviewers screened each record and each report retrieved, whether they worked independently, and if applicable, details of automation tools used in the process.	Page 3
Data collection process	9	Specify the methods used to collect data from reports, including how many reviewers collected data from each report, whether they worked independently, any processes for obtaining or confirming data from study investigators, and if applicable, details of automation tools used in the process.	Page 3
Data items	10a	List and define all outcomes for which data were sought. Specify whether all results that were compatible with each outcome domain in each study were sought (e.g. for all measures, time points, analyses), and if not, the methods used to decide which results to collect.	Page 3
	10b	List and define all other variables for which data were sought (e.g. participant and intervention characteristics, funding sources). Describe any assumptions made about any missing or unclear information.	Page 3
Study risk of bias assessment	11	Specify the methods used to assess risk of bias in the included studies, including details of the tool(s) used, how many reviewers assessed each study and whether they worked independently, and if applicable, details of automation tools used in the process.	Page 3
Effect measures	12	Specify for each outcome the effect measure(s) (e.g. risk ratio, mean difference) used in the synthesis or presentation of results.	Page 3
Synthesis methods	13a	Describe the processes used to decide which studies were eligible for each synthesis (e.g. tabulating the study intervention characteristics and comparing against the planned groups for each synthesis (item #5)).	Page 4
	13b	Describe any methods required to prepare the data for presentation or synthesis, such as handling of missing summary statistics, or data conversions.	Page 4

Section and Topic	Item #	Checklist item	Location where item is reported
	13c	Describe any methods used to tabulate or visually display results of individual studies and syntheses.	Page 4
	13d	Describe any methods used to synthesize results and provide a rationale for the choice(s). If meta-analysis was performed, describe the model(s), method(s) to identify the presence and extent of statistical heterogeneity, and software package(s) used.	Page 4
	13e	Describe any methods used to explore possible causes of heterogeneity among study results (e.g. subgroup analysis, meta-regression).	Page 4
	13f	Describe any sensitivity analyses conducted to assess robustness of the synthesized results.	Page 4
Reporting bias assessment	14	Describe any methods used to assess risk of bias due to missing results in a synthesis (arising from reporting biases).	Page 3
Certainty assessment	15	Describe any methods used to assess certainty (or confidence) in the body of evidence for an outcome.	NA
**RESULTS**			
Study selection	16a	Describe the results of the search and selection process, from the number of records identified in the search to the number of studies included in the review, ideally using a flow diagram.	Page 5
	16b	Cite studies that might appear to meet the inclusion criteria, but which were excluded, and explain why they were excluded.	Page 5
Study characteristics	17	Cite each included study and present its characteristics.	Page 5
Risk of bias in studies	18	Present assessments of risk of bias for each included study.	Page 6
Results of individual studies	19	For all outcomes, present, for each study: (a) summary statistics for each group (where appropriate) and (b) an effect estimate and its precision (e.g. confidence/credible interval), ideally using structured tables or plots.	Page 7-8
Results of syntheses	20a	For each synthesis, briefly summarise the characteristics and risk of bias among contributing studies.	Page 7-8
	20b	Present results of all statistical syntheses conducted. If meta-analysis was done, present for each the summary estimate and its precision (e.g. confidence/credible interval) and measures of statistical heterogeneity. If comparing groups, describe the direction of the effect.	Page 7-8
	20c	Present results of all investigations of possible causes of heterogeneity among study results.	Page 7-8
	20d	Present results of all sensitivity analyses conducted to assess the robustness of the synthesized results.	Page 7-8
Reporting biases	21	Present assessments of risk of bias due to missing results (arising from reporting biases) for each synthesis assessed.	Page 6
Certainty of evidence	22	Present assessments of certainty (or confidence) in the body of evidence for each outcome assessed.	NA
**DISCUSSION**			
Discussion	23a	Provide a general interpretation of the results in the context of other evidence.	Page 13
	23b	Discuss any limitations of the evidence included in the review.	Page 14
	23c	Discuss any limitations of the review processes used.	Page 16

Section and Topic	Item #	Checklist item	Location where item is reported
	23d	Discuss implications of the results for practice, policy, and future research.	Page 13-16
**OTHER INFORMATION**			
Registration and protocol	24a	Provide registration information for the review, including register name and registration number, or state that the review was not registered.	Page 2
	24b	Indicate where the review protocol can be accessed, or state that a protocol was not prepared.	Page 2
	24c	Describe and explain any amendments to information provided at registration or in the protocol.	NA
Support	25	Describe sources of financial or non-financial support for the review, and the role of the funders or sponsors in the review.	Page 17
Competing interests	26	Declare any competing interests of review authors.	Page 17
Availability of data, code and other materials	27	Report which of the following are publicly available and where they can be found: template data collection forms; data extracted from included studies; data used for all analyses; analytic code; any other materials used in the review.	Page 17

Table S2: Search strategies.

Database	Search strategy	Results * 24-11-25
PubMed	((((((((((((((Disuse) OR (Disuse model)) OR (Disuse-induced)) OR (Muscle disuse)) OR (Immobilization)) OR (Immobilized)) OR (Mechanical unloading)) OR (Cast)) OR (Splint)) OR (Slings)) OR (Plaster))) AND ((((((((((((((((Strength) OR (Muscle contraction)) OR (Maximum voluntary contraction)) OR (Maximal voluntary contraction)) OR (One repetition maximum)) OR (Handgrip)) OR (Grip)) OR (Pinch)) OR (Muscle size)) OR (Muscle mass)) OR (Muscle atrophy)) OR (Muscle loss)) OR (Muscle wasting)) OR (Lean body mass)) OR (Cross-sectional area)) OR (Muscle thickness)))) AND ((((((((Shoulder) OR (Elbow)) OR (Wrist)) OR (Hand)) OR (Forearm)) OR (Arm)) OR (Upper limb)))) AND (((((((((Clinical trial) OR (Controlled clinical trial)) OR (Randomized clinical trial)) OR (Cohort Study)) OR (Prospective study)) OR (Pre–post study)) OR (Experimental Study)) OR (Quasi-experimental study))))	1,105
Scopus	(TITLE-ABS-KEY ((Disuse) OR (Disuse model) OR (Disuse-induced) OR (Muscle disuse) OR (Immobilization) OR (Immobilized) OR (Mechanical unloading) OR (Cast) OR (Splint) OR (Slings) OR (Plaster)) AND TITLE-ABS-KEY ((Strength) OR (Muscle contraction) OR (Maximum voluntary contraction) OR (Maximal voluntary contraction) OR (One repetition maximum) OR (Handgrip) OR (Grip) OR (Pinch) OR (Muscle size) OR (Muscle mass) OR (Muscle atrophy) OR (Muscle loss) OR (Muscle wasting) OR (Lean body mass) OR (Cross-sectional area) OR (Muscle thickness)) AND TITLE-ABS-KEY ((Shoulder) OR (Elbow) OR (Wrist) OR (Hand) OR (Forearm) OR (Arm) OR (Upper limb)) AND TITLE-ABS-KEY ((Clinical trial) OR (Controlled clinical trial) OR (Randomized clinical trial) OR (Cohort Study) OR (Prospective study) OR (Pre–post study) OR (Experimental Study) OR (Quasi-experimental study)))	1,274
Web of Science	Results for (Disuse) OR (Disuse model) OR (Disuse-induced) OR (Muscle disuse) OR (Immobilization) OR (Immobilized) OR (Mechanical unloading) OR (Cast) OR (Splint) OR (Slings) OR (Plaster) (All Fields) AND (Strength) OR (Muscle contraction) OR (Maximum voluntary contraction) OR (Maximal voluntary contraction) OR (One repetition maximum) OR (Handgrip) OR (Grip) OR (Pinch) OR (Muscle size) OR (Muscle mass) OR (Muscle atrophy) OR (Muscle loss) OR (Muscle wasting) OR (Lean body mass) OR (Cross-sectional area) OR (Muscle thickness) (All Fields) AND (Shoulder) OR (Elbow) OR (Wrist) OR (Hand) OR (Forearm) OR (Arm) OR (Upper limb) (All Fields) AND (Controlled clinical trial) OR (Randomized clinical trial) OR (Cohort Study) OR (Prospective study) OR (Pre–post study) OR (Experimental Study) OR (Quasi-experimental study) (All Fields)	807
Embase	(disuse OR 'disuse model' OR (disuse AND ('model'/exp OR model)) OR 'disuse induced' OR 'muscle disuse'/exp OR 'muscle disuse' OR (('muscle'/exp OR muscle) AND disuse) OR 'immobilization'/exp OR immobilization OR immobilized OR 'mechanical unloading'/exp OR 'mechanical unloading' OR (mechanical AND unloading) OR 'cast'/exp OR cast OR 'splint'/exp OR splint OR slings OR 'plaster'/exp OR plaster) AND (strength OR (muscle AND contraction) OR (maximum AND voluntary AND contraction) OR	1,438

	(maximal AND voluntary AND contraction) OR (one AND repetition AND maximum) OR handgrip OR grip OR pinch OR (muscle AND size) OR (muscle AND mass) OR (muscle AND atrophy) OR (muscle AND loss) OR (muscle AND wasting) OR (lean AND body AND mass) OR ('cross sectional' AND area) OR (muscle AND thickness)) AND (shoulder OR elbow OR wrist OR hand OR forearm OR arm OR (upper AND limb)) AND (controlled AND clinical AND trial OR (randomized AND clinical AND trial) OR (cohort AND study) OR (prospective AND study) OR (pre–post AND study) OR (experimental AND study) OR ('quasi experimental' AND study))	
Epistemonikos	((((((((((((((Disuse) OR (Disuse model)) OR (Disuse-induced)) OR (Muscle disuse)) OR (Immobilization)) OR (Immobilized)) OR (Mechanical unloading)) OR (Cast)) OR (Splint)) OR (Slings)) OR (Plaster))) AND ((((((((((((((((Strength) OR (Muscle contraction)) OR (Maximum voluntary contraction)) OR (Maximal voluntary contraction)) OR (One repetition maximum)) OR (Handgrip)) OR (Grip)) OR (Pinch)) OR (Muscle size)) OR (Muscle mass)) OR (Muscle atrophy)) OR (Muscle loss)) OR (Muscle wasting)) OR (Lean body mass)) OR (Cross-sectional area)) OR (Muscle thickness)))) AND ((((((((Shoulder) OR (Elbow)) OR (Wrist)) OR (Hand)) OR (Forearm)) OR (Arm)) OR (Upper limb)))) AND (((((((((Clinical trial) OR (Controlled clinical trial)) OR (Randomized clinical trial)) OR (Cohort Study)) OR (Prospective study)) OR (Pre–post study)) OR (Experimental Study)) OR (Quasi-experimental study))))	449
CINAHL	((((((((((((((Disuse) OR (Disuse model)) OR (Disuse-induced)) OR (Muscle disuse)) OR (Immobilization)) OR (Immobilized)) OR (Mechanical unloading)) OR (Cast)) OR (Splint)) OR (Slings)) OR (Plaster))) AND ((((((((((((((((Strength) OR (Muscle contraction)) OR (Maximum voluntary contraction)) OR (Maximal voluntary contraction)) OR (One repetition maximum)) OR (Handgrip)) OR (Grip)) OR (Pinch)) OR (Muscle size)) OR (Muscle mass)) OR (Muscle atrophy)) OR (Muscle loss)) OR (Muscle wasting)) OR (Lean body mass)) OR (Cross-sectional area)) OR (Muscle thickness)))) AND ((((((((Shoulder) OR (Elbow)) OR (Wrist)) OR (Hand)) OR (Forearm)) OR (Arm)) OR (Upper limb)))) AND (((((((((Clinical trial) OR (Controlled clinical trial)) OR (Randomized clinical trial)) OR (Cohort Study)) OR (Prospective study)) OR (Pre–post study)) OR (Experimental Study)) OR (Quasi-experimental study))))	189
SPORTDiscus	((((((((((((((Disuse) OR (Disuse model)) OR (Disuse-induced)) OR (Muscle disuse)) OR (Immobilization)) OR (Immobilized)) OR (Mechanical unloading)) OR (Cast)) OR (Splint)) OR (Slings)) OR (Plaster))) AND ((((((((((((((((Strength) OR (Muscle contraction)) OR (Maximum voluntary contraction)) OR (Maximal voluntary contraction)) OR (One repetition maximum)) OR (Handgrip)) OR (Grip)) OR (Pinch)) OR (Muscle size)) OR (Muscle mass)) OR (Muscle atrophy)) OR (Muscle loss)) OR (Muscle wasting)) OR (Lean body mass)) OR (Cross-sectional area)) OR (Muscle thickness)))) AND ((((((((Shoulder) OR (Elbow)) OR (Wrist)) OR (Hand)) OR (Forearm)) OR (Arm)) OR (Upper limb)))) AND (((((((((Clinical trial) OR (Controlled clinical trial)) OR (Randomized clinical trial)) OR (Cohort Study)) OR (Prospective study)) OR (Pre–post study)) OR (Experimental Study)) OR (Quasi-experimental study))))	54

LILACS	(((((((((((((((Disuse) OR (Disuse model)) OR (Disuse-induced)) OR (Muscle disuse)) OR (Immobilization)) OR (Immobilized)) OR (Mechanical unloading)) OR (Cast)) OR (Splint)) OR (Slings)) OR (Plaster))) AND (((((((((((((((((Strength) OR (Muscle contraction)) OR (Maximum voluntary contraction)) OR (Maximal voluntary contraction)) OR (One repetition maximum)) OR (Handgrip)) OR (Grip)) OR (Pinch)) OR (Muscle size)) OR (Muscle mass)) OR (Muscle atrophy)) OR (Muscle loss)) OR (Muscle wasting)) OR (Lean body mass)) OR (Cross-sectional area)) OR (Muscle thickness)))) AND ((((((((Shoulder) OR (Elbow)) OR (Wrist)) OR (Hand)) OR (Forearm)) OR (Arm)) OR (Upper limb)))) AND (((((((((Clinical trial) OR (Controlled clinical trial)) OR (Randomized clinical trial)) OR (Cohort Study)) OR (Prospective study)) OR (Pre–post study)) OR (Experimental Study)) OR (Quasi-experimental study))))	4
Total		5,320

*The searches were conducted in July 2024, and updated in November 2025.

Table S3: Studies excluded.

	Article	Reason
1	Andrushko JW, Gould LA, Farthing JP. Contralateral effects of unilateral training: sparing of muscle strength and size after immobilization. Appl Physiol Nutr Metab. 2018 Nov;43(11):1131-1139. doi: 10.1139/apnm-2018-0073. Epub 2018 Mar 9. PMID: 29800529.	Wrong design
2	Bassolino M, Campanella M, Bove M, Pozzo T, Fadiga L. Training the motor cortex by observing the actions of others during immobilization. Cereb Cortex. 2014 Dec;24(12):3268-76. doi: 10.1093/cercor/bht190. Epub 2013 Jul 29. PMID: 23897648; PMCID: PMC4224244.	Wrong outcome
3	De Marco D, Scalona E, Bazzini MC, Nuara A, Taglione E, Lopomo NF, Rizzolatti G, Fabbri-Destro M, Avanzini P. Observation of others' actions during limb immobilization prevents the subsequent decay of motor performance. Proc Natl Acad Sci U S A. 2021 Nov 23;118(47):e2025979118. doi: 10.1073/pnas.2025979118. PMID: 34782480; PMCID: PMC8617512.	Wrong outcome
4	Asao A, Nomura T, Shibuya K. Effects of Repetitive Peripheral Magnetic Stimulation through Hand Splint Materials on Induced Movement and Corticospinal Excitability in Healthy Participants. Brain Sci. 2022 Feb 17;12(2):280. doi: 10.3390/brainsci12020280. PMID: 35204043; PMCID: PMC8869939.	No disuse-induced model
5	Villatte J, Taconnat L, Bidet-Ildei C, Toussaint L. Short-term upper limb immobilization and the embodied view of memory: A pilot study. PLoS One. 2021 Mar 11;16(3):e0248239. doi: 10.1371/journal.pone.0248239. PMID: 33705459; PMCID: PMC7951805.	Wrong outcome
6	Pelet DCS, Orsatti FL. Effects of resistance training at different intensities of load on cross-education of muscle strength. Appl Physiol Nutr Metab. 2021 Oct;46(10):1279-1289. doi: 10.1139/apnm-2021-0088. Epub 2021 May 13. PMID: 33984253.	No disuse-induced model
7	Dirks ML, Wall BT, Otten B, Cruz AM, Dunlop MV, Barker AR, Stephens FB. High-fat Overfeeding Does Not Exacerbate Rapid Changes in Forearm Glucose and Fatty Acid Balance During Immobilization. J Clin Endocrinol Metab. 2020 Jan 1;105(1):dgz049. doi: 10.1210/clinem/dgz049. PMID: 31609422.	Wrong outcome
8	Ward GR, MacDougall JD, Sutton JR, Toews CJ, Jones NL. Activation of human muscle pyruvate dehydrogenase with activity and immobilization. Clin Sci (Lond). 1986 Feb;70(2):207-10. doi: 10.1042/cs0700207. PMID: 3956111.	Wrong outcome
9	[Mental practice has influence on limitation of motion and muscle atrophy following immobilisation of the radiocarpal	No disuse-induced model<br>Other language

	joint - a prospective randomised experimental study]. Z Orthop Unfall. 2011 Jun;149(3):288-95. German. doi: 10.1055/s-0030-1270918. Epub 2011 Apr 29. PMID: 21534184.	
10	Frenkel MO, Herzig DS, Gebhard F, Mayer J, Becker C, Einsiedel T. Mental practice maintains range of motion despite forearm immobilization: a pilot study in healthy persons. J Rehabil Med. 2014 Mar;46(3):225-32. doi: 10.2340/16501977-1263. PMID: 24519331.	Wrong outcome
11	Toussaint L, Meugnot A, Bidet-Ildei C. Short-term upper limb immobilisation impairs grasp representation. Q J Exp Psychol (Hove). 2021 Jun;74(6):1096-1102. doi: 10.1177/1747021820985523. Epub 2021 Jan 17. PMID: 33327883.	Wrong outcome
12	Sayers SP, Clarkson PM, Lee J. Activity and immobilization after eccentric exercise: II. Serum CK. Med Sci Sports Exerc. 2000 Sep;32(9):1593-7. doi: 10.1097/00005768-200009000-00011. PMID: 10994910.	Wrong outcome
13	Okamoto Y, Ishii D, Yamamoto S, Ishibashi K, Kohno Y, Numata K. Effects of short-term upper limb immobilization on sensory information processing and corticospinal excitability. Exp Brain Res. 2022 Aug;240(7-8):1979-1989. doi: 10.1007/s00221-022-06371-1. Epub 2022 May 19. PMID: 35589856.	No disuse-induced model
14	Sale DG, McComas AJ, MacDougall JD, Upton AR. Neuromuscular adaptation in human thenar muscles following strength training and immobilization. J Appl Physiol Respir Environ Exerc Physiol. 1982 Aug;53(2):419-24. doi: 10.1152/jappl.1982.53.2.419. PMID: 6288637.	No disuse-induced model
15	de Jong JCBC, Jameson TSO, Andrews RC, Dunlop MV, Abdelrahman DR, Murton AJ, Caspers MPM, Worms N, van Nieuwkoop A, Keijzer N, Cheng Q, Guigas B, van Duijn E, Vaes WHJ, Nieuwenhuizen AG, Keijer J, Wall BT, Verschuren L, Stephens FB, van den Hoek AM, Dirks ML. Anabolic Effects of Salbutamol Are Lost Upon Immobilization. J Cachexia Sarcopenia Muscle. 2025 Dec;16(6):e70114. doi: 10.1002/jcsm.70114.	Wrong outcome

Table S4. Grading of Recommendation, Assessment, Development and Evaluation (GRADE).

Certainty assessment							№ of patients		Effect		Certainty	Importance
**№ of studies**	**Study design**	**Risk of bias**	**Inconsistency**	**Indirectness**	**Imprecision**	**Other considerations**	**Disuse-induced model**		**Relative (95% CI)**	**Absolute (95% CI)**		
**Skeletal muscle mass**												
29	non-randomised studies	serious	not serious	serious	serious	none	251		-	SMD **0.453 SD lower** (0.698 lower to 0.208 lower)	⊕◯◯◯ Very low	NOT IMPORTANT
**Muscle strength**												
33	non-randomised studies	serious	serious	not serious	serious	publication bias strongly suspected	496		-	SMD **1.36 SD lower** (1.69 lower to 1.02 lower)	⊕◯◯◯ Very low	NOT IMPORTANT

CI: confidence interval; **SMD:** standardised mean difference

Table S5: Risk of bias of clinical trials

ITEMS	Q1	Q2	Q3	Q4	Q5	Q6	Q7	Q8	Q9	Q10	Q11	Q12	Q13			
Author & year	Was true randomization used for assignment of participants to treatment groups?	Was allocation to treatment groups concealed?	Were treatment groups similar at the baseline?	Were participants blind to treatment assignment?	Were those delivering treatment blind to treatment assignment?	Were outcomes assessors blind to treatment assignment?	Were treatment groups treated identically other than the intervention of interest?	Was follow up complete and if not, were differences between groups in terms of their follow up adequately described and analyzed?	Were participants analyzed in the groups to which they were randomized?	Were outcomes measured in the same way for treatment groups?	Were outcomes measured in a reliable way?	Was appropriate statistical analysis used?	Was the trial design appropriate, and any deviations from the standard RCT design accounted for in the conduct and analysis of the trial?	Score	%	Risk of Bias
Andrushko et al., 2018	Unclear	Unclear	No	No	No	Yes	Yes	Yes	Unclear	Yes	Yes	Yes	Yes	7	53.85	Moderate
Bostock et al., 2017	Unclear	Unclear	Yes	Yes	Unclear	Unclear	No	Unclear	Unclear	Yes	Yes	Yes	Yes	6	46.15	High
Bostock et al., 2017b	Unclear	Unclear	Yes	Yes	Unclear	Unclear	No	Unclear	Unclear	Yes	Yes	Yes	Yes	6	46.15	High
Carr et al., 2024	Yes	Unclear	No	No	No	Yes	Yes	Yes	Yes	Yes	Unclear	Yes	Yes	8	61.54	Moderate
Chen et al., 2005	Unclear	Unclear	Yes	No	No	No	Yes	Unclear	Unclear	Yes	Unclear	Yes	Yes	5	38.46	High
Gaffney et al., 2021	Yes	Unclear	Yes	No	No	No	Yes	Yes	Unclear	Yes	Unclear	Yes	Yes	7	53.85	Moderate
Homma et al., 2009	Unclear	Unclear	Unclear	No	No	No	Yes	Yes	Unclear	Yes	Unclear	Yes	Yes	5	38.46	High
Homma et al., 2015	Unclear	Unclear	No	No	No	No	Yes	Unclear	Unclear	Yes	Unclear	Yes	Yes	4	30.77	High

Inada et al., 2015	Unclear	Unclear	Yes	No	No	No	Yes	Unclear	Unclear	No	Unclear	Yes	Yes	4	30.77	High
Johnston et al., 2009	Unclear	Unclear	Unclear	Yes	No	No	No	Unclear	Unclear	Yes	Unclear	Yes	Yes	4	30.77	High
Matsumura et al., 2008	Unclear	Unclear	No	No	No	No	Yes	Unclear	Unclear	Yes	Unclear	Yes	Yes	4	30.77	High
Motobe et al., 2004	Unclear	Unclear	No	No	No	No	Yes	Unclear	Unclear	Yes	Unclear	Yes	Yes	4	30.77	High
Newsom et al., 2003	Unclear	Unclear	No	No	No	No	Yes	Unclear	Unclear	Yes	Unclear	Yes	Yes	4	30.77	High
Ngomo et al., 2012	Unclear	Unclear	Unclear	No	No	No	Yes	Unclear	Unclear	Yes	Unclear	Yes	Yes	4	30.77	High
Ohmori et al., 2010	Unclear	Unclear	Unclear	No	No	No	Yes	Unclear	Unclear	Yes	Unclear	Yes	Yes	4	30.77	High
Pearce et al., 2013	Unclear	Unclear	No	No	No	No	Yes	Unclear	Unclear	Yes	Yes	Yes	Yes	5	38.46	High
Rittweger et al., 2005	Unclear	Unclear	No	No	No	No	No	Unclear	Unclear	Yes	Unclear	Yes	Yes	3	23.08	High
Sayers et al., 2000	Unclear	Unclear	No	No	No	No	Yes	Unclear	Unclear	Yes	Yes	Yes	Yes	5	38.46	High
Ulloa-Escalante et al., 2022	Yes	Unclear	No	No	No	No	Yes	Unclear	Unclear	Yes	Unclear	Yes	Yes	5	38.46	High
Valdes et al., 2021	Unclear	Unclear	No	No	No	No	Yes	Unclear	Unclear	Yes	Yes	Yes	Yes	5	38.46	High
Zainuddin et al., 2005	Unclear	Unclear	Unclear	No	No	No	Yes	Unclear	Unclear	Yes	Unclear	Yes	Yes	4	30.77	High

JBI critical appraisal for clinical trials. Q1: Was true randomization used for assignment of participants to treatment groups? Q2: Was allocation to treatment groups concealed? Q3 Were treatment groups similar at the baseline? Q4: Were participants blind to treatment assignment? Q5: Were those delivering treatment blind to treatment assignment? Q6: Were outcomes assessors blind to treatment assignment? Q7: Were treatment groups treated identically other than the intervention of interest? Q8: Was follow up complete and if not, were differences between groups in terms of their follow up adequately described and analyzed? Q9: Were participants analyzed in the groups to which they were randomized? Q10: Were outcomes measured in the same way for treatment groups? Q11: Were outcomes measured in a reliable way? Q12: Was appropriate statistical analysis used? Q13: Was the trial design appropriate, and any deviations from the standard RCT design accounted for in the conduct and analysis of the trial?

Table S6: Risk of bias of cuasi-experimental studies

ITEM	Q1	Q2	Q3	Q4	Q5	Q6	Q7	Q8	Q9			
Author & year	Is it clear in the study what is the ‘cause’ and what is the ‘effect’ (i.e. there is no confusion about which variable comes first)?	Were the participants included in any comparisons similar?	Were the participants included in any comparisons receiving similar treatment, other than the exposure or intervention of interest?	Was there a control group?	Were there multiple measurements of the outcome both pre and post the intervention/exposure?	Was follow up complete and if not, were differences between groups in terms of their follow up adequately described and analyzed?	Were the outcomes of participants included in any comparisons measured in the same way?	Were outcomes measured in a reliable way?	Was appropriate statistical analysis used?	Score	%	Risk of Bias
Boer et al. 2008	Yes	Yes	Yes	No	No	Unclear	Yes	Yes	Yes	6	66.67	Moderate
Chen et al. 2022	Yes	Yes	No	Yes	No	Yes	Yes	Yes	Yes	7	77.78	Low
Chen et al., 2023	Yes	Yes	Yes	Yes	No	Yes	Yes	Yes	Yes	8	88.89	Low
Clark et al., 2008	Yes	Unclear	No	Yes	No	Unclear	Yes	No	Yes	4	44.44	High
Clark et al., 2009	Yes	Unclear	No	No	No	Unclear	Yes	No	Yes	3	33.33	High
Clark et al., 2010	Yes	Unclear	No	Yes	No	Yes	Yes	No	Yes	5	55.56	Moderate
Clark et al. 2014	Yes	No	Yes	Yes	No	Unclear	Yes	Yes	Yes	6	66.67	Moderate
Farthing et al., 2009	Yes	No	No	Yes	No	Unclear	Yes	No	Yes	4	44.44	High
Farthing et al., 2011	Yes	No	No	Yes	Yes	Yes	Yes	No	Yes	6	66.67	Moderate
Fuglevand et al. 1995	Yes	Yes	No	No	No	Unclear	Yes	No	Yes	4	44.44	High
Karolczak et al, 2009	Yes	No	No	Yes	No	Unclear	Yes	No	Yes	4	44.44	High
Kitahara et al., 2003	Yes	Yes	No	No	No	Unclear	Yes	No	Yes	4	44.44	High
Lundbye-jensen-nielsen 2008	Yes	Yes	No	No	No	Unclear	Yes	No	Yes	4	44.44	High
MacIntyre et al., 2001	Yes	Yes	Yes	Yes	No	No	Yes	No	Yes	6	66.67	Moderate

Magnus et al., 2010	Yes	No	Yes	Yes	No	Unclear	Yes	No	Yes	5	55.56	Moderate
Miles et al., 2005	Yes	No	No	Yes	No	Yes	Yes	No	Yes	5	55.56	Moderate
Parcell et al. 2000	Yes	Yes	No	No	No	Unclear	Yes	No	Yes	4	44.44	High
Seki et al. 2001	Yes	Yes	No	No	No	Unclear	Yes	No	Yes	4	44.44	High
Seki et al. 2007	Yes	Yes	No	No	No	Yes	Yes	No	Yes	5	55.56	Moderate
Semmler et al., 1999	Yes	No	No	Yes	No	Unclear	Yes	No	Yes	4	44.44	High
Stock et al. 2025	Yes	Yes	Yes	Yes	Yes	Yes	Yes	Yes	Yes	9	100	Low
Urso et al, 2006	Yes	No	No	No	Yes	Yes	Yes	No	Yes	5	55.56	Moderate
Vaughan 1989	Yes	Yes	No	no	Yes	Unclear	Yes	Yes	Yes	6	66.67	Moderate
Yue et al. 1997	Yes	Yes	No	No	Yes	Unclear	Yes	Yes	Yes	6	66.67	Moderate

JBI critical appraisal for cuasi-experimental studies. Q1: Is it clear in the study what is the ‘cause’ and what is the ‘effect’ (i.e. there is no confusion about which variable comes first)? Q2: Were the participants included in any comparisons similar? Q3: Were the participants included in any comparisons receiving similar treatment, other than the exposure or intervention of interest? Q4: Was there a control group? Q5: Were there multiple measurements of the outcome both pre and post the intervention/exposure? Q6: Was follow up complete and if not, were differences between groups in terms of their follow up adequately described and analyzed? Q7: Were the outcomes of participants included in any comparisons measured in the same way? Q8: Were outcomes measured in a reliable way? Q9: Was appropriate statistical analysis used?

Figure S1: Forest plot skeletal muscle mass.

Random-Effects Model (k = 29)

	Estimate	se	Z	p	CI Lower Bound	CI Upper Bound
Intercept	-0.453	0.120	-3.79	< .001	-0.698	-0.208
	.	.	.	.	.	.

Nota. Tau² Estimator: Sidik-Jonkman. Knapp and Hartung (2003) adjustment used.

[3]

Heterogeneity Statistics

Tau	Tau²	I²	H²	R²	df	Q	p
0.496	0.2457 (SE= 0.0805)	49.6%	1.984	.	28.000	47.855	0.011

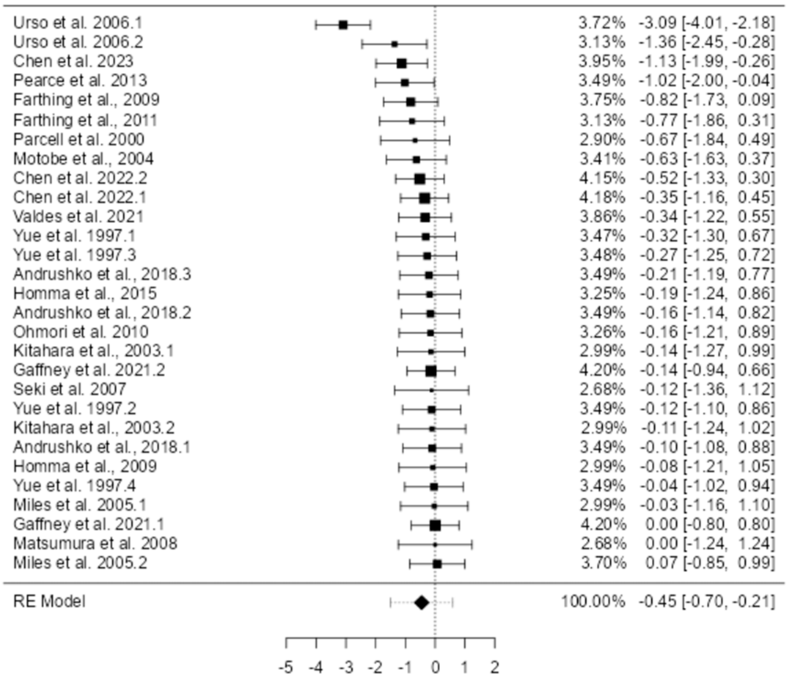

Figure S2: Funnel plot skeletal muscle mass.

Publication Bias Assessment

Test Name	value	p
Fail-Safe N	223.000	< .001
Begg and Mazumdar Rank Correlation	-0.034	0.809
Egger's Regression	0.714	0.481
Trim and Fill Number of Studies	8.000	.

Nota. Fail-safe N Calculation Using the Rosenthal Approach

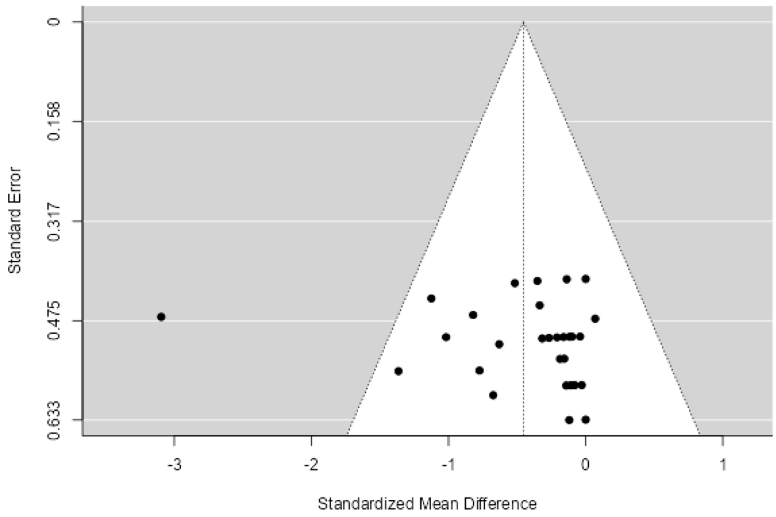

Figure S3: Sensitivity analysis. Forest plot skeletal muscle mass.

Random-Effects Model (k = 28)

	Estimate	se	Z	p	CI Lower Bound	CI Upper Bound
Intercept	-0.353	0.0706	-5.00	< .001	-0.498	-0.208
	.	.	.	.	.	.

Nota. Tau² Estimator: Sidik-Jonkman. Knapp and Hartung (2003) adjustment used.

[3]

Heterogeneity Statistics

Tau	Tau²	I²	H²	R²	df	Q	p
0.221	0.0488 (SE= 0.0289)	16.3%	1.195	.	27.000	14.993	0.970

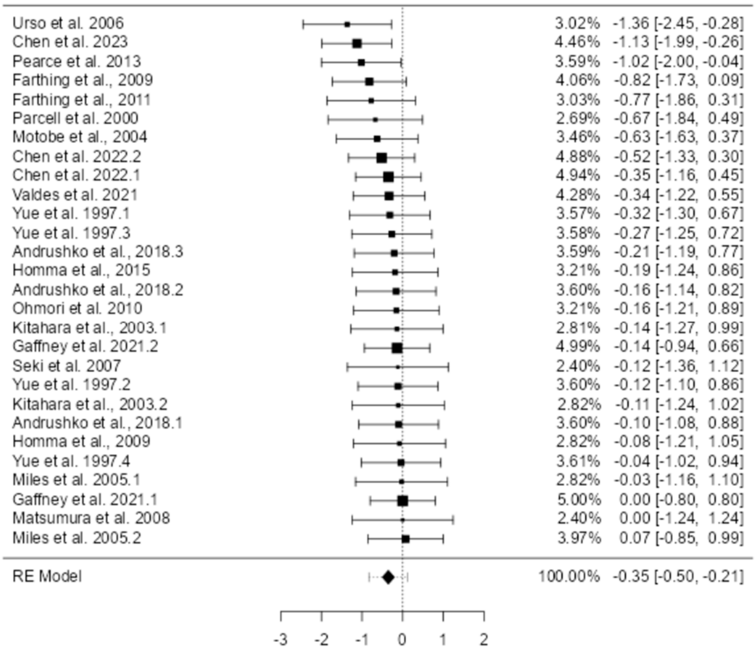

Figure S4: Sensitivity analysis. Funnel plot skeletal muscle mass.

Publication Bias Assessment

Test Name	value	p
Fail-Safe N	113.000	< .001
Begg and Mazumdar Rank Correlation	-0.042	0.769
Egger's Regression	0.353	0.727
Trim and Fill Number of Studies	4.000	.

Nota. Fail-safe N Calculation Using the Rosenthal Approach

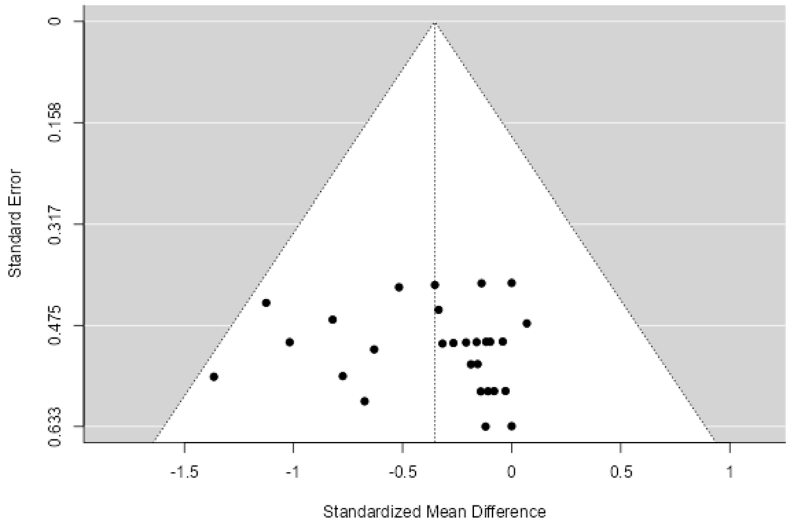

Figure S5: Sensitivity analysis that includes only imaging studies. Forest plot skeletal muscle mass.

Random-Effects Model (k = 20)

	Estimate	se	Z	p	CI Lower Bound	CI Upper Bound
Intercept	−0.560	0.167	−3.35	0.003	−0.909	−0.210
	.	.	.	.	.	.

Nota. Tau² Estimator: Sidik-Jonkman. Knapp and Hartung (2003) adjustment used.

[3]

Heterogeneity Statistics

Tau	Tau²	I²	H²	R²	df	Q	p
0.603	0.363 (SE= 0.1339)	58.34%	2.400	.	19.000	43.129	0.001

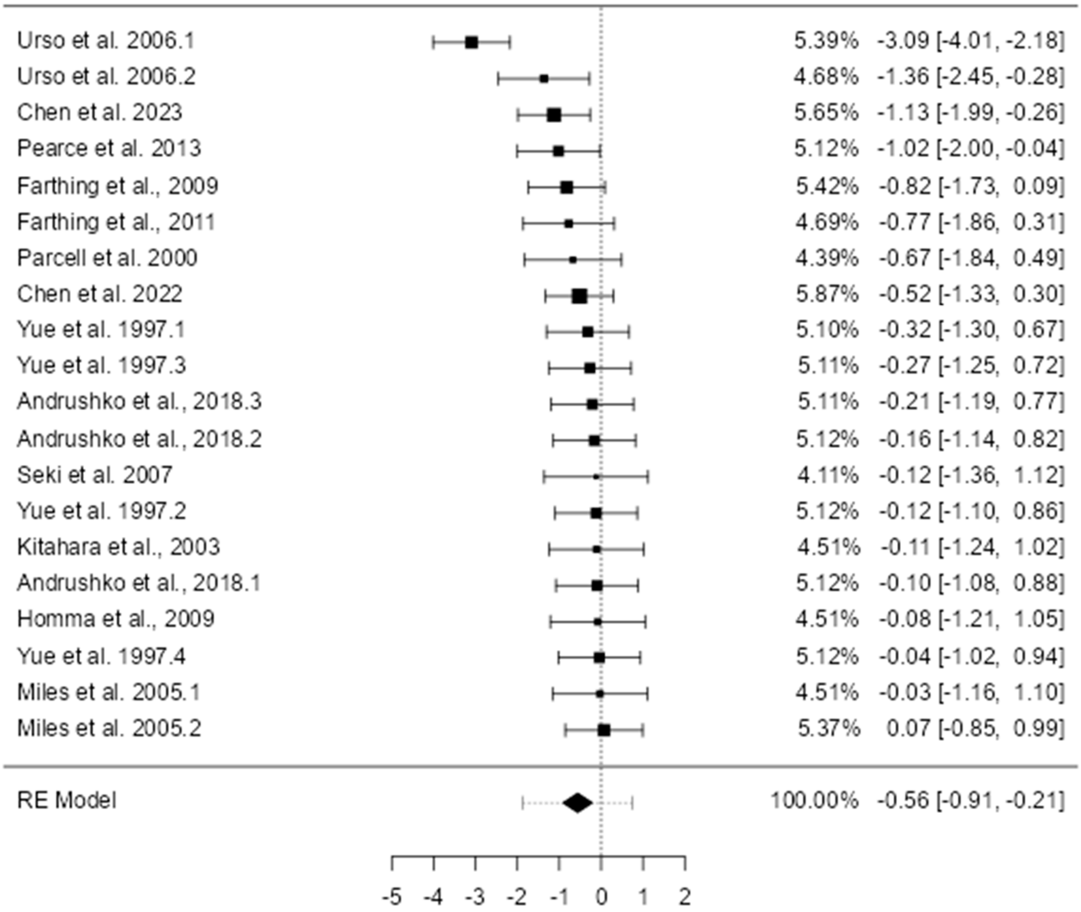

Figure S6: Sensitivity analysis that includes only imaging studies. Funnel plot skeletal muscle mass.

Publication Bias Assessment

Test Name	value	p
Fail-Safe N	160.000	< .001
Begg and Mazumdar Rank Correlation	−0.032	0.873
Egger's Regression	1.136	0.271
Trim and Fill Number of Studies	6.000	.

Nota. Fail-safe N Calculation Using the Rosenthal Approach

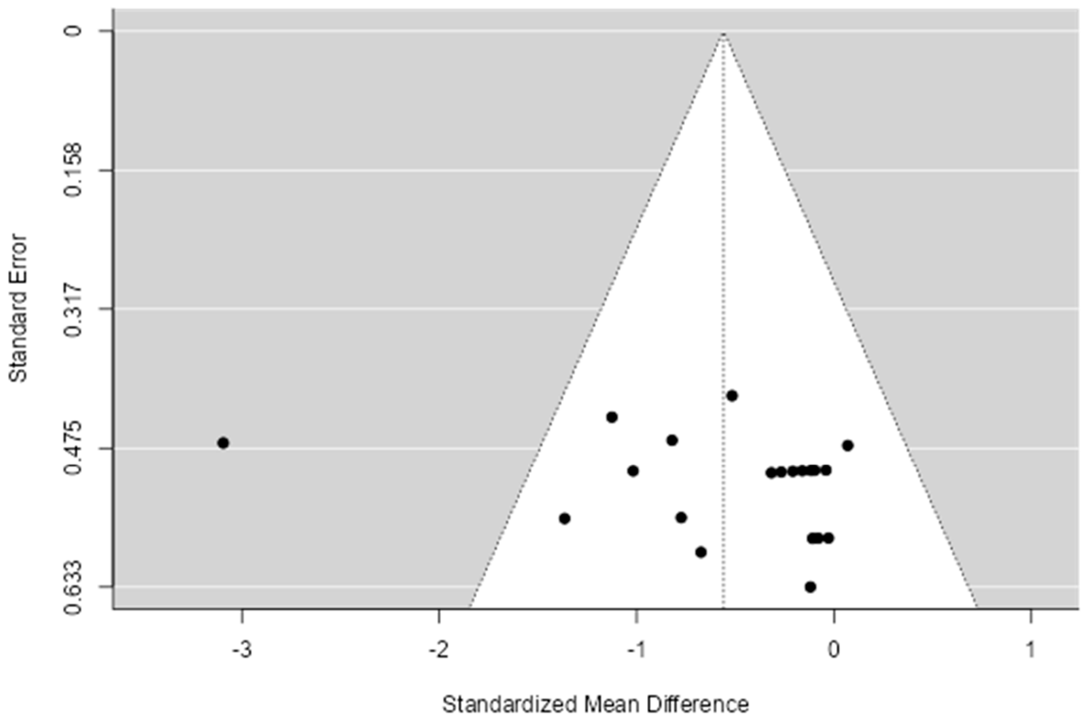

Figure S7: Forest plot skeletal muscle strength.

Random-Effects Model (k = 55)

	Estimate	se	Z	p	CI Lower Bound	CI Upper Bound
Intercept	−1.36	0.168	−8.07	< .001	−1.696	−1.021
	.	.	.	.	.	.

Nota. Tau² Estimator: Sidik-Jonkman. Knapp and Hartung (2003) adjustment used.

[3]

Heterogeneity Statistics

Tau	Tau²	I²	H²	R²	df	Q	p
1.172	1.3732 (SE= 0.2778)	82.76%	5.802	.	54.000	187.806	< .001

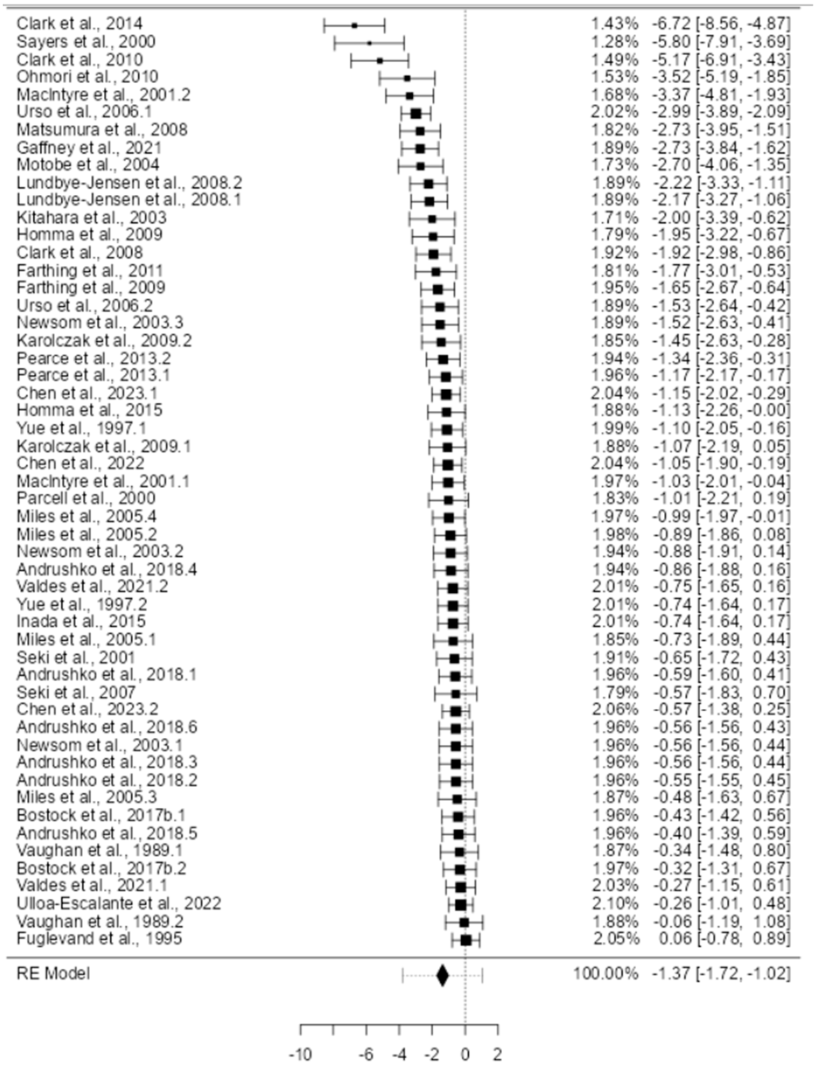

Figure S8: Funnel plot skeletal muscle strength.

Publication Bias Assessment

Test Name	value	p
Fail-Safe N	5664.000	< .001
Begg and Mazumdar Rank Correlation	-0.451	< .001
Egger's Regression	-8.883	< .001
Trim and Fill Number of Studies	0.000	.

Nota. Fail-safe N Calculation Using the Rosenthal Approach

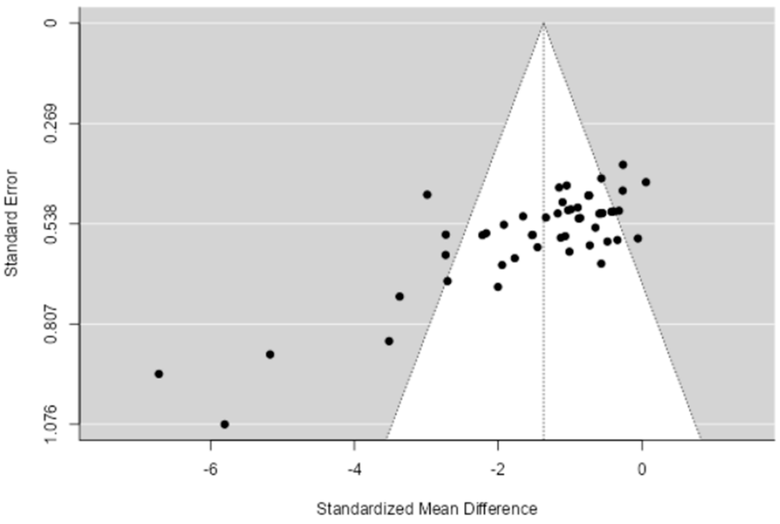

Figure S9: Sensitivity analysis. Forest plot skeletal muscle strength.

Random-Effects Model (k = 52)

	Estimate	se	Z	p	CI Lower Bound	CI Upper Bound
Intercept	−1.14	0.113	−10.1	< .001	−1.369	−0.914
	.	.	.	.	.	.

Nota. Tau² Estimator: Sidik-Jonkman. Knapp and Hartung (2003) adjustment used.

[3]

Heterogeneity Statistics

Tau	Tau²	I²	H²	R²	df	Q	p
0.695	0.4835 (SE= 0.1093)	63.75%	2.759	.	51.000	112.327	< .001

Forest Plot

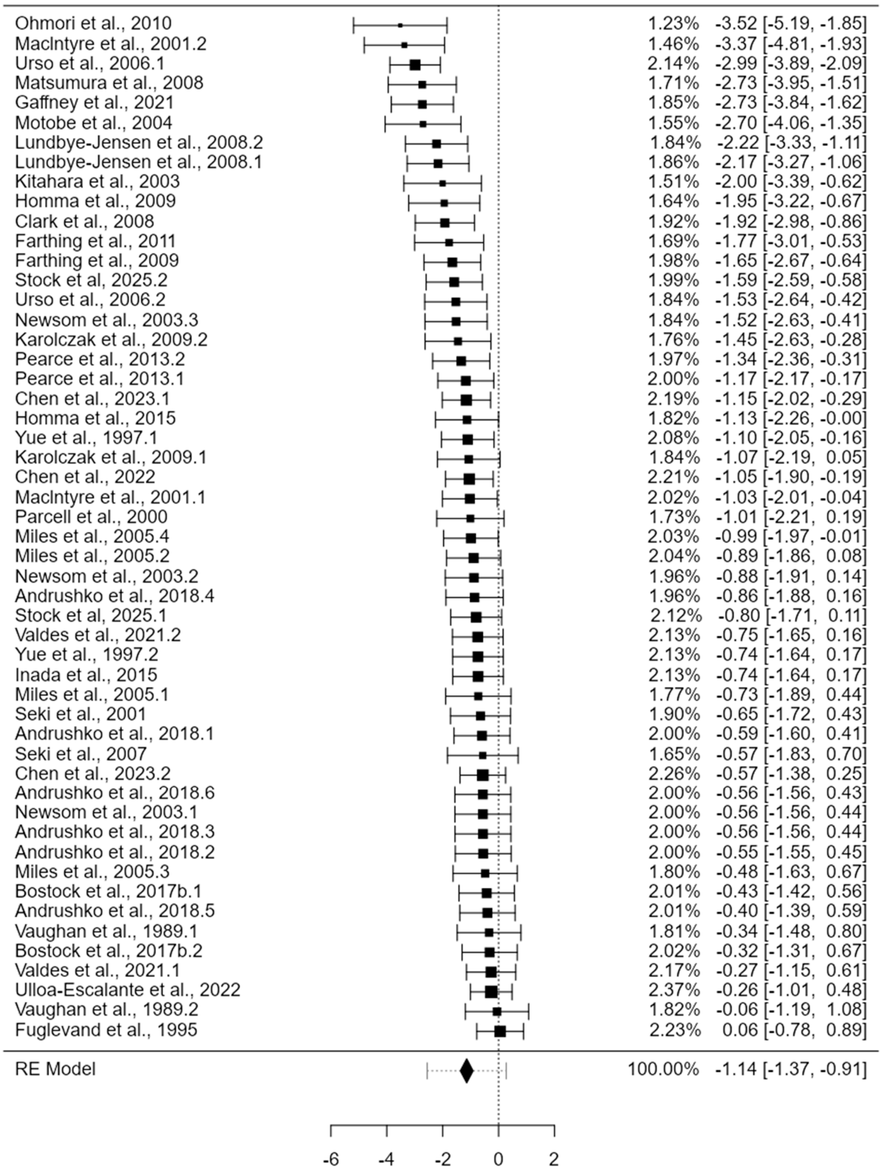

Figure S10: Sensitivity analysis. Funnel plot skeletal muscle strength.

Publication Bias Assessment

Test Name	value	p
Fail-Safe N	4488.000	< .001
Begg and Mazumdar Rank Correlation	-0.371	< .001
Egger's Regression	-4.911	< .001
Trim and Fill Number of Studies	0.000	.

Nota. Fail-safe N Calculation Using the Rosenthal Approach

Funnel Plot

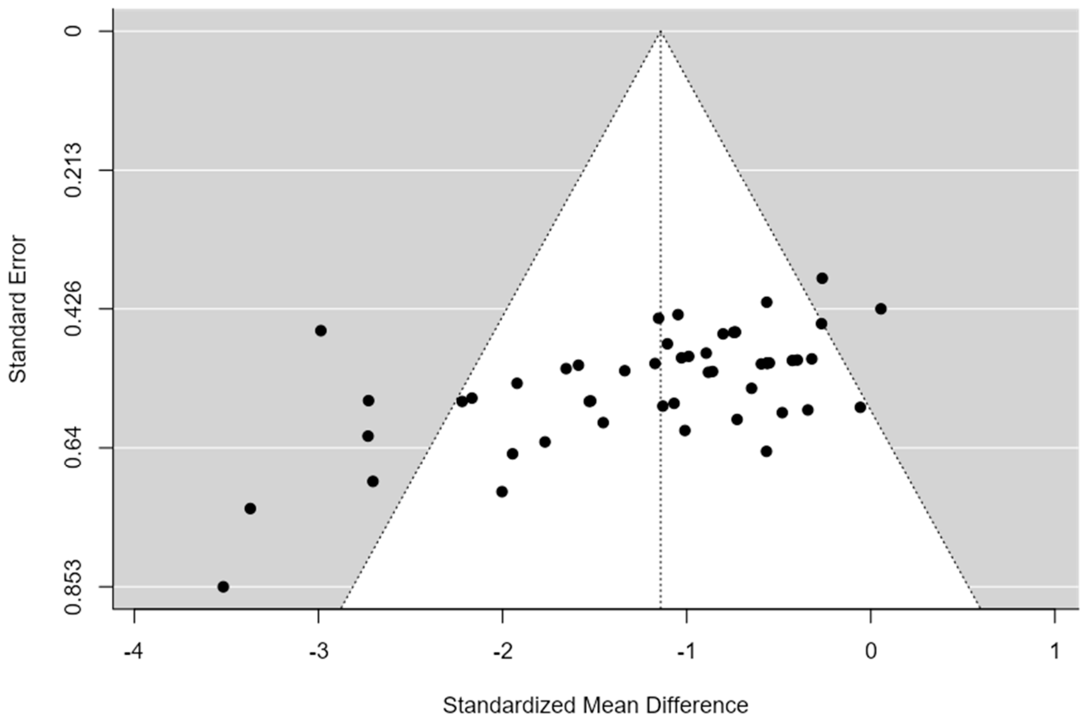

Supplement: Supplementary file 1 [file jcm-14-08884-s001.zip › jcm-3996035-supplementary.pdf]
